# Supplementary material for: Associations Between Polygenic Risk for Alzheimer’s Disease and Grey Matter Volume Are Dependent on APOE, Pathological and Diagnostic Status
Source: Genes (Basel). 2025 Sep 25;16(10):1128. doi: 10.3390/genes16101128 (PMC12562698; doi:10.3390/genes16101128)
Supplement: Supplementary file 1 [file genes-16-01128-s001.zip › genes-3837032-supplementary.pdf]

genes

Supplementary materials

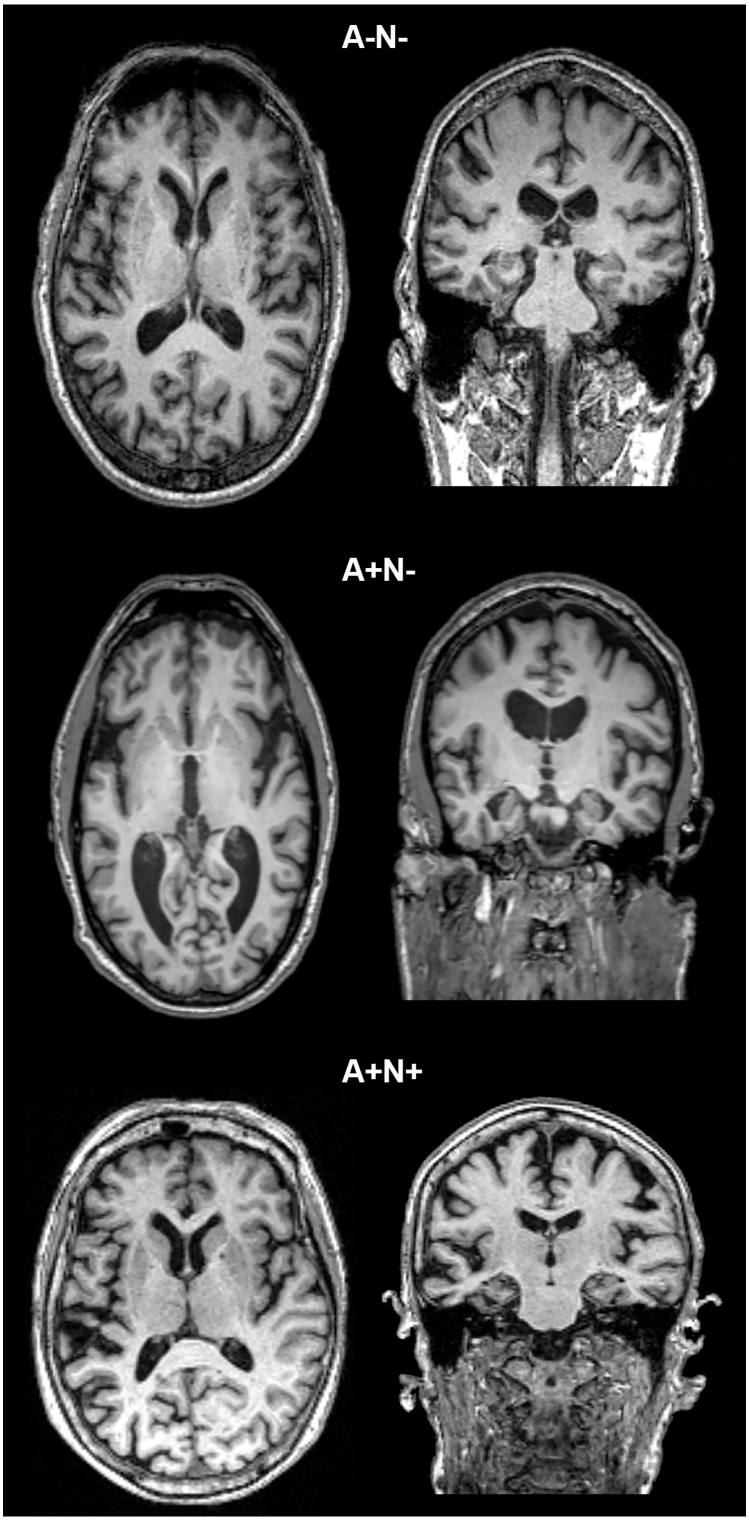

Figure S1. Axial and coronal T1 weighted structural images of a typical participant for each group, selected based on the group-specific median grey matter fraction (GMF) values: A-N- is a 74 y/o cognitively unimpaired male, with 12 years of education and ε4 non-carrier (GMF = 0.421); A+N- is a 71 y/o male with AD, 20 years of education and ε4 carrier (GMF = 0.403); A+N+ is a 82 y/o female with AD, 20 years of education and ε4 non-carrier (GMF = 0.346).

Table S1. Significant associations between PRS1 and regional grey matter volumes in the whole sample. Values are β ± SE and associated p-values from regression models.

	PRS1	Age	Sex	Education	TIV	MF
L AMY	**-0.037±0.006, <0.001**	**-0.005±0.001, <0.001**	**0.049±0.016, 0.001**	0.001±0.002, 0.753	**0.000±0.000, <0.001**	**0.026±0.012, 0.029**
R AMY	**-0.032±0.006, <0.001**	**-0.005±0.001, <0.001**	**0.047±0.016, 0.002**	-0.001±0.002, 0.701	**0.000±0.000, <0.001**	**0.028±0.012, 0.019**
L HIP	**-0.126±0.020, <0.001**	**-0.015±0.003, <0.001**	**0.208±0.056, <0.001**	-0.004±0.008, 0.625	**0.002±0.000, <0.001**	**0.125±0.042, 0.003**
R HIP	**-0.134±0.020, <0.001**	**-0.016±0.003, <0.001**	**0.181±0.054, 0.001**	0.000±0.007, 0.977	**0.002±0.000, <0.001**	**0.154±0.042, <0.001**
L PHG	**-0.140±0.022, <0.001**	**-0.018±0.003, <0.001**	**0.215±0.060, <0.001**	0.003±0.008, 0.698	**0.001±0.000, <0.001**	**0.113±0.046, 0.014**
R PHG	**-0.125±0.022, <0.001**	**-0.014±0.003, <0.001**	**0.235±0.059, <0.001**	-0.001±0.008, 0.916	**0.002±0.000, <0.001**	**0.167±0.045, <0.001**
L MTG	**-0.496±0.093, <0.001**	**-0.078±0.015, <0.001**	0.435±0.255, 0.088	-0.037±0.035, 0.283	**0.007±0.001, <0.001**	**0.423±0.194, 0.029**
R MTG	**-0.496±0.083, <0.001**	**-0.067±0.008, <0.001**	0.295±0.228, 0.195	-0.002±0.031, 0.939	**0.007±0.001, <0.001**	**0.545±0.174, 0.002**
L STG	**-0.228±0.049, <0.001**	**-0.053±0.008, <0.001**	0.174±0.133, 0.188	-0.019±0.018, 0.299	**0.003±0.000, <0.001**	**0.207±0.101, 0.040**
R STG	**-0.292±0.061, <0.001**	**-0.062±0.010, <0.001**	0.230±0.166, 0.166	0.003±0.023, 0.899	**0.004±0.001, <0.001**	**0.340±0.127, 0.007**
L FG	**-0.282±0.050, <0.001**	**-0.038±0.008, <0.001**	**0.547±0.138, <0.001**	-0.002±0.019, 0.908	**0.004±0.000, <0.001**	**0.310±0.105, 0.003**
R FG	**-0.222±0.043, <0.001**	**-0.044±0.007, <0.001**	**0.618±0.118, <0.001**	-0.014±0.016, 0.374	**0.005±0.000, <0.001**	**0.460±0.090, <0.001**
L mPFC	**-0.056±0.013, <0.001**	**-0.009±0.002, <0.001**	0.023±0.037, 0.536	-0.006±0.005, 0.202	**0.001±0.000, <0.001**	-0.035±0.028,0.216
R mPFC	**-0.066±0.017, <0.001**	**-0.009±0.003, 0.001**	0.035±0.046, 0.438	-0.003±0.006, 0.620	**0.001±0.000, <0.001**	-0.012±0.035,0.723
L PCC	**-0.061±0.011, <0.001**	**-0.010±0.002, <0.001**	0.047±0.029, 0.105	0.000±0.004, 0.964	**0.001±0.000, <0.001**	**0.050±0.022, 0.024**
R PCC	**-0.038±0.007, <0.001**	**-0.004±0.001, <0.001**	**0.059±0.020, 0.003**	-0.001±0.003, 0.638	**0.000±0.000, <0.001**	**0.037±0.015, 0.014**

L: Left; R: Right; AMY: Amygdala; FG: Fusiform gyrus; HIP: Hippocampus; mPFC: medial pre-frontal cortex; MF: magnetic field; MTG: middle temporal gyrus; PHG: Parahippocampal gyrus; PCC: posterior cingulate cortex; STG: superior temporal gyrus; TIV: total intracranial volume.

The table reports only results for those brain regions where at least one predictor was significantly associated with grey matter volume.

In bold: significant associations between predictors and regional volumes.

Table S2. Significant associations between PRS2 and regional grey matter volumes in the whole sample. Values are β (SE) and associated p-values from regression models.

	PRS2	Age	Sex	Education	TIV	MF
L AMY	**-0.036±0.006, <0.001**	**-0.005±0.001, <0.001**	**0.049±0.016, 0.002**	0.001±0.002, 0.742	**0.000±0.000, <0.001**	**0.026±0.012, 0.032**
R AMY	**-0.031±0.006, <0.001**	**-0.005±0.001, <0.001**	**0.047±0.015, 0.002**	-0.001±0.002, 0.715	**0.000±0.000, <0.001**	**0.028±0.012, 0.018**
L HIP	**-0.124±0.020, <0.001**	**-0.015±0.003, <0.001**	**0.207±0.055, <0.001**	-0.004±0.008, 0.613	**0.002±0.000, <0.001**	**0.125±0.042, 0.003**
R HIP	**-0.132±0.020, <0.001**	**-0.016±0.003, <0.001**	**0.180±0.054, 0.001**	0.000±0.007, 0.994	**0.002±0.000, <0.001**	**0.154±0.041, <0.001**
L PHG	**-0.137±0.022, <0.001**	**-0.017±0.003, <0.001**	**0.214±0.060, <0.001**	0.003±0.008, 0.686	**0.001±0.000, <0.001**	**0.113±0.046, 0.014**
R PHG	**-0.123±0.022, <0.001**	**-0.014±0.003, <0.001**	**0.233±0.060, <0.001**	-0.001±0.008, 0.927	**0.002±0.000, <0.001**	**0.167±0.045, <0.001**
L MTG	**-0.487±0.093, <0.001**	**-0.078±0.015, <0.001**	0.434±0.254, 0.088	-0.038±0.035, 0.279	**0.007±0.001, <0.001**	**0.425±0.194, 0.029**
R MTG	**-0.484±0.085, <0.001**	**-0.067±0.013, <0.001**	0.291±0.231, 0.208	-0.002±0.032, 0.956	**0.007±0.001, <0.001**	**0.545±0.176, 0.002**
L STG	**-0.223±0.049, <0.001**	**-0.053±0.008, <0.001**	0.172±0.133, 0.197	-0.019±0.018, 0.305	**0.003±0.000, <0.001**	**0.208±0.101, 0.040**
R STG	**-0.286±0.061, <0.001**	**-0.062±0.010, <0.001**	0.226±0.166, 0.173	0.003±0.023, 0.883	**0.004±0.001, <0.001**	**0.341±0.127, 0.007**
L FG	**-0.274±0.050, <0.001**	**-0.038±0.008, <0.001**	**0.545±0.138, <0.001**	-0.002±0.019, 0.913	**0.004±0.000, <0.001**	**0.309±0.105, 0.003**
R FG	**-0.217±0.043, <0.001**	**-0.044±0.007, <0.001**	**0.616±0.118, <0.001**	-0.014±0.016, 0.387	**0.005±0.000, <0.001**	**0.459±0.090, <0.001**
L mPFC	**-0.054±0.014, <0.001**	**-0.009±0.002, <0.001**	0.022±0.037, 0.556	-0.006±0.005, 0.206	**0.001±0.000, <0.001**	-0.035±0.028,0.218
R mPFC	**-0.065±0.017, <0.001**	**-0.009±0.003, 0.001**	0.034±0.046, 0.456	-0.003±0.006, 0.626	**0.001±0.000, <0.001**	-0.012±0.035,0.725
L PCC	**-0.059±0.011, <0.001**	**-0.010±0.002, <0.001**	0.047±0.029, 0.106	0.000±0.004, 0.978	**0.001±0.000, <0.001**	**0.050±0.022, 0.024**
R PCC	**-0.037±0.007, <0.001**	**-0.004±0.001, <0.001**	**0.059±0.020, 0.003**	-0.001±0.003, 0.653	**0.000±0.000, <0.001**	**0.037±0.015, 0.015**

L: Left; R: Right; AMY: Amygdala; FG: Fusiform gyrus; HIP: Hippocampus; mPFC: medial pre-frontal cortex; MF: magnetic field; MTG: middle temporal gyrus; PHG: Parahippocampal gyrus; PCC: posterior cingulate cortex; STG: superior temporal gyrus; TIV: total intracranial volume.

The table reports only results for those brain regions where at least one predictor was significantly associated with grey matter volume.

In bold: significant associations between predictors and regional volumes.

Table S3. ROI-wise associations between AD PRS and regional grey matter volume with joint FDR (q-values) across ROIs, groups, and PRSs.

	Group	PRS	β	SE	N	p	q
**Left Amygdala**	A+N+	PRS1	-0.025	0.010	114	0.018	0.458
**Left Amygdala**	A+N+	PRS2	-0.025	0.010	114	0.019	0.458
**Right Amygdala**	A+N+	PRS1	-0.007	0.008	114	0.404	0.809
**Right Amygdala**	A+N+	PRS2	-0.007	0.008	114	0.429	0.841
**Left HIP**	A+N+	PRS1	-0.051	0.032	114	0.122	0.584
**Left HIP**	A+N+	PRS2	-0.053	0.032	114	0.102	0.584
**Right HIP**	A+N+	PRS1	-0.053	0.028	114	0.061	0.584
**Right HIP**	A+N+	PRS2	-0.055	0.028	114	0.054	0.577
**Left PHG**	A+N+	PRS1	-0.067	0.031	114	0.034	0.539
**Left PHG**	A+N+	PRS2	-0.068	0.031	114	0.031	0.539
**Right PHG**	A+N+	PRS1	-0.037	0.029	114	0.201	0.650
**Right PHG**	A+N+	PRS2	-0.038	0.029	114	0.187	0.650
**Left MTG**	A+N+	PRS1	-0.060	0.090	114	0.507	0.889
**Left MTG**	A+N+	PRS2	-0.059	0.090	114	0.509	0.889
**Right MTG**	A+N+	PRS1	-0.019	0.096	114	0.846	0.946
**Right MTG**	A+N+	PRS2	-0.014	0.096	114	0.885	0.946
**Left STG**	A+N+	PRS1	-0.007	0.052	114	0.893	0.946
**Left STG**	A+N+	PRS2	-0.005	0.052	114	0.918	0.946
**Right STG**	A+N+	PRS1	0.071	0.070	114	0.308	0.705
**Right STG**	A+N+	PRS2	0.075	0.070	114	0.287	0.671
**Left FG**	A+N+	PRS1	-0.017	0.057	114	0.772	0.946
**Left FG**	A+N+	PRS2	-0.014	0.057	114	0.805	0.946
**Right FG**	A+N+	PRS1	0.031	0.055	114	0.572	0.894
**Right FG**	A+N+	PRS2	0.031	0.055	114	0.572	0.894
**Left mPFC**	A+N+	PRS1	0.010	0.016	114	0.548	0.892

**Left mPFC**	A+N+	PRS2	0.012	0.016	114	0.461	0.869
**Right mPFC**	A+N+	PRS1	0.024	0.021	114	0.264	0.650
**Right mPFC**	A+N+	PRS2	0.026	0.021	114	0.234	0.650
**Left PCC**	A+N+	PRS1	-0.022	0.013	114	0.101	0.584
**Left PCC**	A+N+	PRS2	-0.021	0.013	114	0.118	0.584
**Right PCC**	A+N+	PRS1	-0.005	0.007	114	0.483	0.874
**Right PCC**	A+N+	PRS2	-0.005	0.007	114	0.519	0.890
**Left Amygdala**	A-N-	PRS1	-0.001	0.010	114	0.923	0.946
**Left Amygdala**	A-N-	PRS2	-0.001	0.010	114	0.909	0.946
**Right Amygdala**	A-N-	PRS1	-0.006	0.009	114	0.541	0.892
**Right Amygdala**	A-N-	PRS2	-0.005	0.009	114	0.578	0.894
**Left HIP**	A-N-	PRS1	0.008	0.034	114	0.822	0.946
**Left HIP**	A-N-	PRS2	0.010	0.033	114	0.763	0.946
**Right HIP**	A-N-	PRS1	0.004	0.034	114	0.896	0.946
**Right HIP**	A-N-	PRS2	0.005	0.033	114	0.887	0.946
**Left PHG**	A-N-	PRS1	0.023	0.037	114	0.533	0.892
**Left PHG**	A-N-	PRS2	0.026	0.037	114	0.480	0.874
**Right PHG**	A-N-	PRS1	0.034	0.037	114	0.359	0.768
**Right PHG**	A-N-	PRS2	0.035	0.037	114	0.342	0.764
**Left MTG**	A-N-	PRS1	-0.055	0.169	114	0.747	0.946
**Left MTG**	A-N-	PRS2	-0.061	0.168	114	0.716	0.946
**Right MTG**	A-N-	PRS1	-0.124	0.137	114	0.368	0.768
**Right MTG**	A-N-	PRS2	-0.124	0.136	114	0.363	0.768
**Left STG**	A-N-	PRS1	-0.100	0.086	114	0.248	0.650
**Left STG**	A-N-	PRS2	-0.110	0.085	114	0.198	0.650
**Right STG**	A-N-	PRS1	-0.123	0.103	114	0.236	0.650
**Right STG**	A-N-	PRS2	-0.129	0.102	114	0.211	0.650
**Left FG**	A-N-	PRS1	-0.009	0.087	114	0.915	0.946

**Left FG**	A-N-	PRS2	-0.008	0.086	114	0.929	0.946
**Right FG**	A-N-	PRS1	-0.041	0.077	114	0.595	0.906
**Right FG**	A-N-	PRS2	-0.035	0.076	114	0.648	0.942
**Left mPFC**	A-N-	PRS1	-0.009	0.026	114	0.717	0.946
**Left mPFC**	A-N-	PRS2	-0.011	0.026	114	0.668	0.946
**Right mPFC**	A-N-	PRS1	-0.007	0.029	114	0.804	0.946
**Right mPFC**	A-N-	PRS2	-0.009	0.029	114	0.752	0.946
**Left PCC**	A-N-	PRS1	-0.033	0.019	114	0.081	0.584
**Left PCC**	A-N-	PRS2	-0.033	0.018	114	0.076	0.584
**Right PCC**	A-N-	PRS1	-0.045	0.015	114	0.003	0.160
**Right PCC**	A-N-	PRS2	-0.046	0.014	114	0.002	0.160
**Left Amygdala**	A+N-	PRS1	-0.011	0.009	114	0.206	0.650
**Left Amygdala**	A+N-	PRS2	-0.010	0.009	114	0.256	0.650
**Right Amygdala**	A+N-	PRS1	-0.011	0.009	114	0.210	0.650
**Right Amygdala**	A+N-	PRS2	-0.011	0.009	114	0.226	0.650
**Left HIP**	A+N-	PRS1	-0.041	0.026	114	0.115	0.584
**Left HIP**	A+N-	PRS2	-0.037	0.026	114	0.150	0.650
**Right HIP**	A+N-	PRS1	-0.061	0.029	114	0.042	0.574
**Right HIP**	A+N-	PRS2	-0.058	0.029	114	0.049	0.577
**Left PHG**	A+N-	PRS1	-0.047	0.030	114	0.118	0.584
**Left PHG**	A+N-	PRS2	-0.044	0.030	114	0.146	0.650
**Right PHG**	A+N-	PRS1	-0.041	0.031	114	0.187	0.650
**Right PHG**	A+N-	PRS2	-0.040	0.031	114	0.194	0.650
**Left MTG**	A+N-	PRS1	0.043	0.122	114	0.722	0.946
**Left MTG**	A+N-	PRS2	0.058	0.119	114	0.626	0.940
**Right MTG**	A+N-	PRS1	-0.131	0.115	114	0.258	0.650
**Right MTG**	A+N-	PRS2	-0.124	0.115	114	0.284	0.671
**Left STG**	A+N-	PRS1	0.015	0.062	114	0.806	0.946

**Left STG**	A+N-	PRS2	0.025	0.061	114	0.683	0.946
**Right STG**	A+N-	PRS1	-0.075	0.085	114	0.382	0.781
**Right STG**	A+N-	PRS2	-0.068	0.087	114	0.439	0.843
**Left FG**	A+N-	PRS1	-0.025	0.052	114	0.639	0.942
**Left FG**	A+N-	PRS2	-0.020	0.052	114	0.697	0.946
**Right FG**	A+N-	PRS1	-0.087	0.052	114	0.097	0.584
**Right FG**	A+N-	PRS2	-0.084	0.052	114	0.108	0.584
**Left mPFC**	A+N-	PRS1	-0.003	0.020	114	0.877	0.946
**Left mPFC**	A+N-	PRS2	-0.002	0.020	114	0.904	0.946
**Right mPFC**	A+N-	PRS1	0.000	0.025	114	0.990	0.990
**Right mPFC**	A+N-	PRS2	0.002	0.025	114	0.936	0.946
**Left PCC**	A+N-	PRS1	0.002	0.015	114	0.909	0.946
**Left PCC**	A+N-	PRS2	0.003	0.015	114	0.819	0.946
**Right PCC**	A+N-	PRS1	0.012	0.010	114	0.251	0.650
**Right PCC**	A+N-	PRS2	0.013	0.011	114	0.222	0.650

FG: Fusiform gyrus; HIP: Hippocampus; mPFC: medial pre-frontal cortex; MTG: middle temporal gyrus; PHG: Parahippocampal gyrus; PCC: posterior cingulate cortex; STG: superior temporal gyrus.

β: standardised regression coefficient for PRS; SE: robust standard error (HC3) of β; N: number of participants included in the model for that row; p: two-sided raw p-value for the PRS coefficient; q: BH-FDR-adjusted p-value (q-value) computed jointly across ROIs, groups, and PRSs (m=96), significance threshold $q < 0.05$.

Table S4. Significant associations between PRSs without APOE and regional grey matter volumes within individual groups.

	**$PRS1_{noAPOE}$**			**$PRS2_{noAPOE}$**		
	**β**	**SE**	***p***	**β**	**SE**	***p***
**A+N+**						
Left amygdala	-0.025	0.012	0.037	n.s.	n.s.	n.s.
**A-N-**						
Right middle temporal gyrus	-0.174	0.084	0.039	-0.173	0.085	0.043
Left superior temporal gyrus	-0.111	0.053	0.036	-0.117	0.053	0.028
Right superior temporal gyrus	-0.162	0.062	0.009	-0.167	0.062	0.007
Left medial prefrontal cortex	-0.040	0.015	0.009	-0.043	0.015	0.005
Right medial prefrontal cortex	-0.040	0.018	0.032	-0.044	0.019	0.018
Left posterior cingulate cortex	n.s.	n.s.	n.s.	-0.024	0.012	0.040

n.s.: Not significant SE: Standard error

Table S5. Significant associations between PRSs without APOE and regional grey matter volumes within groups stratified by APOE genotype.

	**$PRS1_{noAPOE}$**			**$PRS2_{noAPOE}$**		
	**β**	**SE**	***p***	**β**	**SE**	***p***
**A-N- non-carriers**						
Left superior temporal gyrus	-0.142	0.059	0.016	-0.146	0.058	0.012
Right superior temporal gyrus	-0.192	0.069	0.005	-0.192	0.071	0.007
Left medial prefrontal cortex	-0.037	0.017	0.026	-0.040	0.016	0.013
Right medial prefrontal cortex	-0.042	0.020	0.038	-0.045	0.020	0.025
**A-N- carriers**						
Left amygdala	0.050	0.000	<0.001	0.066	0.000	<0.001
Right amygdala	0.022	0.000	<0.001	0.029	0.000	<0.001
Left hippocampus	0.072	0.000	<0.001	0.086	0.001	<0.001
Rignt hippocampus	-0.039	0.000	<0.001	-0.063	0.001	<0.001
Left parahippocampal gyrus	0.081	0.000	<0.001	0.097	0.001	<0.001
Right parahippocampal gyrus	0.031	0.000	<0.001	0.026	0.001	<0.001
Left middle temporal gyrus	0.448	0.000	<0.001	0.591	0.000	<0.001
Right middle temporal gyrus	0.134	0.000	<0.001	0.177	0.000	<0.001
Left superior temporal gyrus	0.343	0.003	<0.001	0.443	0.000	<0.001
Right superior temporal gyrus	0.239	0.000	<0.001	0.316	0.000	<0.001
Left fusiform gyrus	0.234	0.002	<0.001	0.304	0.000	<0.001
Right fusiform gyrus	0.018	0.000	<0.001	0.024	0.000	<0.001
Left medial prefrontal cortex	0.017	0.004	<0.001	n.s.	n.s.	n.s.
Right medial prefrontal cortex	0.069	0.004	<0.001	0.085	0.007	<0.001
Left posterior cingulate cortex	-0.089	0.000	<0.001	-0.112	0.003	<0.001
Right posterior cingulate cortex	0.014	0.000	<0.001	0.017	0.000	<0.001
**A+N- carriers**						
Left fusiform gyrus	0.204	0.080	0.010	0.191	0.086	0.027
Right fusiform gyrus	0.202	0.082	0.014	0.198	0.083	0.017

n.s.: Not significant, SE: Standard error

Table S6. Significant associations between PRS1$_{noAPOE}$ and regional grey matter volumes in the whole sample. Values are β ± SE and associated p-values from regression models.

	**PRS1$_{noAPOE}$**	**Age**	**Sex**	**Education**	**TIV**	**MF**
**L AMY**	**-0.019±0.006, 0.002**	**-0.005±0.001, <0.001**	**0.042±0.016, 0.011**	0.001±0.002, 0.528	**0.000±0.000, <0.001**	**0.027±0.013, 0.032**
**R AMY**	**-0.014±0.006, 0.015**	**-0.005±0.001, <0.001**	**0.039±0.016, 0.014**	0.000±0.002, 0.904	**0.000±0.000, <0.001**	**0.032±0.012, 0.009**
**L HIP**	**-0.068±0.021, 0.001**	**-0.014±0.003, <0.001**	**0.177±0.058, 0.002**	0.006±0.008, 0.420	**0.002±0.000, <0.001**	**0.137±0.044, 0.002**
**R HIP**	**-0.065±0.021, 0.002**	**-0.015±0.003, <0.001**	**0.141±0.058, 0.014**	0.003±0.008, 0.732	**0.002±0.000, <0.001**	**0.166±0.044, <0.001**
**L PHG**	**-0.070±0.023, 0.003**	**-0.016±0.004, <0.001**	**0.179±0.064, 0.005**	0.006±0.009, 0.492	**0.001±0.000, <0.001**	**0.127±0.049, 0.009**
**R PHG**	**-0.052±0.023, 0.024**	**-0.013±0.004, <0.001**	**0.200±0.063, 0.002**	0.002±0.009, 0.838	**0.002±0.000, <0.001**	**0.182±0.048, <0.001**
**L MTG**	**-0.231±0.097, 0.017**	**-0.076±0.015, <0.001**	0.324±0.263, 0.219	-0.027±0.036, 0.455	**0.007±0.001, <0.001**	**0.490±0.200, 0.015**
**R MTG**	**-0.231±0.087, 0.008**	**-0.063±0.014, <0.001**	0.143±0.237, 0.547	0.010±0.032, 0.748	**0.007±0.001, <0.001**	**0.613±0.180, 0.001**
**L STG**	**-0.119±0.049, 0.015**	**-0.052±0.008, <0.001**	0.121±0.134, 0.367	-0.014±0.018, 0.449	**0.003±0.000, <0.001**	**0.229±0.102, 0.025**
**R STG**	**-0.151±0.064, 0.018**	**-0.061±0.010, <0.001**	0.149±0.174, 0.391	0.010±0.024, 0.666	**0.004±0.001, <0.001**	**0.381±0.132, 0.004**
**L FG**	-0.103±0.053, 0.054	**-0.036±0.008, <0.001**	**0.485±0.146, 0.001**	0.004±0.020, 0.827	**0.004±0.000, <0.001**	**0.337±0.111, 0.002**
**R FG**	**-0.093±0.045, 0.036**	**-0.042±0.007, <0.001**	**0.579±0.122, <0.001**	-0.010±0.017, 0.564	**0.005±0.000, <0.001**	**0.484±0.092, <0.001**
**L mPFC**	**-0.035±0.014, 0.009**	**-0.009±0.002, <0.001**	0.011±0.037, 0.764	-0.005±0.005, 0.339	**0.001±0.000, <0.001**	-0.028±0.028,0.332
**R mPFC**	-0.031±0.017, 0.074	**-0.009±0.003, 0.001**	0.016±0.047, 0.732	-0.001±0.006, 0.851	**0.001±0.000, <0.001**	-0.004±0.036,0.907
**L PCC**	-0.020±0.011, 0.077	**-0.010±0.002, <0.001**	0.037±0.030, 0.226	0.001±0.004, 0.841	**0.001±0.000, <0.001**	**0.059±0.023, 0.011**
**R PCC**	**-0.017±0.008, 0.030**	**-0.004±0.001, 0.001**	**0.048±0.021, 0.021**	0.000±0.003, 0.991	**0.000±0.000, <0.001**	**0.041±0.016, 0.010**

L: Left; R: Right; AMY: Amygdala; FG: Fusiform gyrus; HIP: Hippocampus; mPFC: medial pre-frontal cortex; MTG: middle temporal gyrus; PHG: Parahippocampal gyrus; PCC: posterior cingulate cortex; STG: superior temporal gyrus; MF: magnetic field; TIV: total intracranial volume.

The table reports only results for those brain regions where at least one predictor was significantly associated with grey matter volume.

In bold: significant associations between predictors and regional volumes.

Table S7. Significant associations between PRS1$_{noAPOE}$ and regional grey matter volumes in the whole sample. Values are β ± SE and associated p-values from regression models.

	**PRS2$_{noAPOE}$**	**Age**	**Sex**	**Education**	**TIV**	**MF**
**L AMY**	**-0.016±0.006, 0.009**	**-0.005±0.001, <0.001**	**0.042±0.017, 0.011**	0.001±0.002, 0.531	**0.000±0.000, <0.001**	**0.027±0.013, 0.032**
**R AMY**	**-0.012±0.006, 0.045**	**-0.005±0.001, <0.001**	**0.040±0.016, 0.013**	0.000±0.002, 0.920	**0.000±0.000, <0.001**	**0.032±0.012, 0.010**
**L HIP**	**-0.060±0.021, 0.005**	**-0.013±0.003, <0.001**	**0.179±0.058, 0.002**	0.006±0.008, 0.413	**0.002±0.000, <0.001**	**0.138±0.044, 0.002**
**R HIP**	**-0.057±0.021, 0.007**	**-0.015±0.003, <0.001**	**0.144±0.058, 0.013**	0.003±0.008, 0.740	**0.002±0.000, <0.001**	**0.167±0.044, <0.001**
**L PHG**	**-0.061±0.024, 0.011**	**-0.016±0.004, <0.001**	**0.182±0.065, 0.005**	0.006±0.009, 0.490	**0.001±0.000, <0.001**	**0.128±0.049, 0.010**
**R PHG**	-0.045±0.023, 0.051	**-0.013±0.004, 0.001**	**0.202±0.063, 0.001**	0.002±0.009, 0.832	**0.002±0.000, <0.001**	**0.182±0.048, <0.001**
**L MTG**	**-0.204±0.097, 0.036**	**-0.075±0.015, <0.001**	0.331±0.265, 0.210	-0.027±0.036, 0.458	**0.007±0.001, <0.001**	**0.491±0.201, 0.015**
**R MTG**	**-0.211±0.088, 0.016**	**-0.062±0.014, <0.001**	0.144±0.238, 0.547	0.011±0.033, 0.742	**0.007±0.001, <0.001**	**0.612±0.181, 0.001**
**L STG**	**-0.104±0.050, 0.038**	**-0.052±0.008, <0.001**	0.124±0.136, 0.362	-0.014±0.019, 0.452	**0.003±0.000, <0.001**	**0.230±0.104, 0.026**
**R STG**	**-0.139±0.064, 0.030**	**-0.060±0.010, <0.001**	0.152±0.175, 0.383	0.010±0.024, 0.666	**0.004±0.001, <0.001**	**0.382±0.133, 0.004**
**L FG**	-0.082±0.054, 0.128	**-0.036±0.008, <0.001**	**0.491±0.147, 0.001**	0.004±0.020, 0.830	**0.004±0.000, <0.001**	**0.337±0.112, 0.003**
**R FG**	-0.078±0.045, 0.083	**-0.042±0.007, <0.001**	**0.582±0.122, <0.001**	-0.009±0.017, 0.574	**0.005±0.000, <0.001**	**0.483±0.093, <0.001**
**L mPFC**	**-0.035±0.014, 0.011**	**-0.009±0.002, <0.001**	0.012±0.037, 0.753	-0.005±0.005, 0.353	**0.001±0.000, <0.001**	-0.028±0.028,0.317
**R mPFC**	-0.029±0.017, 0.098	**-0.009±0.003, 0.001**	0.016±0.047, 0.732	-0.001±0.006, 0.857	**0.001±0.000, <0.001**	-0.004±0.036,0.904
**L PCC**	-0.016±0.011, 0.154	**-0.010±0.002, <0.001**	0.037±0.030, 0.217	0.001±0.004, 0.850	**0.001±0.000, <0.001**	**0.059±0.023, 0.011**
**R PCC**	-0.014±0.008, 0.070	**-0.004±0.001, 0.001**	**0.049±0.021, 0.018**	0.000±0.003, 0.988	**0.000±0.000, <0.001**	**0.041±0.016, 0.009**

L: Left; R: Right; AMY: Amygdala; FG: Fusiform gyrus; HIP: Hippocampus; mPFC: medial pre-frontal cortex; MTG: middle temporal gyrus; PHG: Parahippocampal gyrus; PCC: posterior cingulate cortex; STG: superior temporal gyrus; MF: magnetic field; TIV: total intracranial volume.

The table reports only results for those brain regions where at least one predictor was significantly associated with grey matter volume.

In bold: significant associations between predictors and regional volumes.

Table S8. Significant associations between PRSs without APOE and brain regional grey matter volumes stratified by diagnosis and APOE carrier status

	$PRS1_{noAPOE}$			$PRS2_{noAPOE}$		
	β	SE	*p*	β	SE	*p*
**CU non-carrier**						
Left superior temporal gyrus	n.s.	n.s.	n.s.	-0.161	0.071	0.024
Right superior temporal gyrus	n.s.	n.s.	n.s.	-0.196	0.087	0.024
**CU carrier**						
Left amygdala	n.s.	n.s.	n.s.	0.018	0.009	0.046
Right amygdala	0.047	0.015	0.001	0.043	0.014	0.002
Right parahippocampal	0.195	0.071	0.006	0.190	0.056	0.001
Left middle temporal gyrus	n.s.	n.s.	n.s.	0.527	0.265	0.046
Right middle temporal gyrus	0.735	0.265	0.006	0.675	0.192	<0.001
Right superior temporal gyrus	0.596	0.146	<0.001	0.566	0.124	<0.001
Left fusiform gyrus	0.491	0.180	0.006	0.396	0.191	0.038
Right fusiform gyrus	0.542	0.139	<0.001	0.521	0.130	<0.001
Left medial prefrontal cortex	0.132	0.055	0.017	n.s.	n.s.	n.s.
Right medial prefrontal cortex	0.180	0.065	0.006	0.155	0.061	0.010
Left posterior cingulate cortex	n.s.	n.s.	n.s.	0.110	0.044	0.012
Right posterior cingulate cortex	0.059	0.026	0.024	0.062	0.021	0.004

n.s.: Not significant; SE: Standard error.

Table S9. ROI-wise associations between AD PRS without APOE load and regional grey matter volume with joint FDR (q-values) across ROIs, groups, and PRSs.

	Group	PRS	β	SE	N	p	q
**Left Amygdala**	A+N+	$PRS1_{noAPOE}$	-0.025	0.012	114	0.040	0.366
**Left Amygdala**	A+N+	$PRS2_{noAPOE}$	-0.022	0.012	114	0.068	0.439
**Right Amygdala**	A+N+	$PRS1_{noAPOE}$	-0.008	0.010	114	0.393	0.649
**Right Amygdala**	A+N+	$PRS2_{noAPOE}$	-0.007	0.010	114	0.481	0.684
**Left HIP**	A+N+	$PRS1_{noAPOE}$	-0.064	0.037	114	0.081	0.439
**Left HIP**	A+N+	$PRS2_{noAPOE}$	-0.060	0.035	114	0.091	0.462
**Right HIP**	A+N+	$PRS1_{noAPOE}$	-0.043	0.032	114	0.181	0.582
**Right HIP**	A+N+	$PRS2_{noAPOE}$	-0.045	0.031	114	0.157	0.582
**Left PHG**	A+N+	$PRS1_{noAPOE}$	-0.066	0.036	114	0.074	0.439
**Left PHG**	A+N+	$PRS2_{noAPOE}$	-0.060	0.036	114	0.097	0.467
**Right PHG**	A+N+	$PRS1_{noAPOE}$	-0.025	0.035	114	0.476	0.684
**Right PHG**	A+N+	$PRS2_{noAPOE}$	-0.027	0.034	114	0.426	0.649
**Left MTG**	A+N+	$PRS1_{noAPOE}$	-0.120	0.101	114	0.236	0.582
**Left MTG**	A+N+	$PRS2_{noAPOE}$	-0.112	0.100	114	0.264	0.617
**Right MTG**	A+N+	$PRS1_{noAPOE}$	-0.043	0.108	114	0.690	0.779
**Right MTG**	A+N+	$PRS2_{noAPOE}$	-0.052	0.107	114	0.631	0.758
**Left STG**	A+N+	$PRS1_{noAPOE}$	-0.040	0.057	114	0.483	0.684
**Left STG**	A+N+	$PRS2_{noAPOE}$	-0.038	0.056	114	0.506	0.684
**Right STG**	A+N+	$PRS1_{noAPOE}$	0.011	0.079	114	0.891	0.910
**Right STG**	A+N+	$PRS2_{noAPOE}$	-0.002	0.077	114	0.978	0.978
**Left FG**	A+N+	$PRS1_{noAPOE}$	-0.013	0.064	114	0.835	0.878
**Left FG**	A+N+	$PRS2_{noAPOE}$	0.013	0.065	114	0.844	0.878
**Right FG**	A+N+	$PRS1_{noAPOE}$	0.027	0.062	114	0.662	0.772
**Right FG**	A+N+	$PRS2_{noAPOE}$	0.030	0.062	114	0.629	0.758
**Left mPFC**	A+N+	$PRS1_{noAPOE}$	0.007	0.019	114	0.707	0.790

**Left mPFC**	A+N+	$PRS2_{noAPOE}$	0.010	0.018	114	0.575	0.723
**Right mPFC**	A+N+	$PRS1_{noAPOE}$	0.031	0.023	114	0.187	0.582
**Right mPFC**	A+N+	$PRS2_{noAPOE}$	0.030	0.024	114	0.205	0.582
**Left PCC**	A+N+	$PRS1_{noAPOE}$	0.016	0.016	114	0.306	0.624
**Left PCC**	A+N+	$PRS2_{noAPOE}$	0.017	0.016	114	0.296	0.618
**Right PCC**	A+N+	$PRS1_{noAPOE}$	0.002	0.009	114	0.803	0.866
**Right PCC**	A+N+	$PRS2_{noAPOE}$	0.004	0.009	114	0.679	0.776
**Left Amygdala**	A-N-	$PRS1_{noAPOE}$	-0.007	0.006	114	0.260	0.617
**Left Amygdala**	A-N-	$PRS2_{noAPOE}$	-0.006	0.006	114	0.325	0.629
**Right Amygdala**	A-N-	$PRS1_{noAPOE}$	-0.006	0.006	114	0.290	0.618
**Right Amygdala**	A-N-	$PRS2_{noAPOE}$	-0.005	0.006	114	0.424	0.649
**Left HIP**	A-N-	$PRS1_{noAPOE}$	-0.016	0.021	114	0.455	0.683
**Left HIP**	A-N-	$PRS2_{noAPOE}$	-0.015	0.021	114	0.495	0.684
**Right HIP**	A-N-	$PRS1_{noAPOE}$	-0.025	0.021	114	0.223	0.582
**Right HIP**	A-N-	$PRS2_{noAPOE}$	-0.025	0.021	114	0.224	0.582
**Left PHG**	A-N-	$PRS1_{noAPOE}$	-0.016	0.023	114	0.502	0.684
**Left PHG**	A-N-	$PRS2_{noAPOE}$	-0.013	0.023	114	0.572	0.723
**Right PHG**	A-N-	$PRS1_{noAPOE}$	-0.015	0.023	114	0.522	0.696
**Right PHG**	A-N-	$PRS2_{noAPOE}$	-0.014	0.023	114	0.558	0.723
**Left MTG**	A-N-	$PRS1_{noAPOE}$	-0.133	0.104	114	0.204	0.582
**Left MTG**	A-N-	$PRS2_{noAPOE}$	-0.146	0.103	114	0.156	0.582
**Right MTG**	A-N-	$PRS1_{noAPOE}$	-0.174	0.084	114	0.042	0.366
**Right MTG**	A-N-	$PRS2_{noAPOE}$	-0.173	0.085	114	0.046	0.366
**Left STG**	A-N-	$PRS1_{noAPOE}$	-0.111	0.053	114	0.038	0.366
**Left STG**	A-N-	$PRS2_{noAPOE}$	-0.117	0.053	114	0.031	0.366
**Right STG**	A-N-	$PRS1_{noAPOE}$	-0.162	0.062	114	0.010	0.249
**Right STG**	A-N-	$PRS2_{noAPOE}$	-0.167	0.062	114	0.008	0.249
**Left FG**	A-N-	$PRS1_{noAPOE}$	-0.029	0.053	114	0.580	0.723

**Left FG**	A-N-	$PRS2_{noAPOE}$	-0.028	0.053	114	0.597	0.734
**Right FG**	A-N-	$PRS1_{noAPOE}$	-0.068	0.049	114	0.169	0.582
**Right FG**	A-N-	$PRS2_{noAPOE}$	-0.061	0.050	114	0.231	0.582
**Left mPFC**	A-N-	$PRS1_{noAPOE}$	-0.040	0.015	114	0.010	0.249
**Left mPFC**	A-N-	$PRS2_{noAPOE}$	-0.043	0.015	114	0.006	0.249
**Right mPFC**	A-N-	$PRS1_{noAPOE}$	-0.040	0.018	114	0.034	0.366
**Right mPFC**	A-N-	$PRS2_{noAPOE}$	-0.044	0.019	114	0.020	0.366
**Left PCC**	A-N-	$PRS1_{noAPOE}$	-0.023	0.012	114	0.054	0.397
**Left PCC**	A-N-	$PRS2_{noAPOE}$	-0.024	0.012	114	0.043	0.366
**Right PCC**	A-N-	$PRS1_{noAPOE}$	-0.016	0.009	114	0.082	0.439
**Right PCC**	A-N-	$PRS2_{noAPOE}$	-0.017	0.009	114	0.072	0.439
**Left Amygdala**	A+N-	$PRS1_{noAPOE}$	0.004	0.009	114	0.657	0.772
**Left Amygdala**	A+N-	$PRS2_{noAPOE}$	0.008	0.009	114	0.364	0.646
**Right Amygdala**	A+N-	$PRS1_{noAPOE}$	0.008	0.009	114	0.358	0.646
**Right Amygdala**	A+N-	$PRS2_{noAPOE}$	0.012	0.009	114	0.186	0.582
**Left HIP**	A+N-	$PRS1_{noAPOE}$	0.001	0.029	114	0.971	0.978
**Left HIP**	A+N-	$PRS2_{noAPOE}$	0.012	0.028	114	0.667	0.772
**Right HIP**	A+N-	$PRS1_{noAPOE}$	0.008	0.030	114	0.786	0.866
**Right HIP**	A+N-	$PRS2_{noAPOE}$	0.020	0.029	114	0.489	0.684
**Left PHG**	A+N-	$PRS1_{noAPOE}$	0.006	0.030	114	0.851	0.878
**Left PHG**	A+N-	$PRS2_{noAPOE}$	0.017	0.030	114	0.570	0.723
**Right PHG**	A+N-	$PRS1_{noAPOE}$	0.026	0.031	114	0.396	0.649
**Right PHG**	A+N-	$PRS2_{noAPOE}$	0.033	0.031	114	0.282	0.618
**Left MTG**	A+N-	$PRS1_{noAPOE}$	0.122	0.125	114	0.334	0.629
**Left MTG**	A+N-	$PRS2_{noAPOE}$	0.136	0.124	114	0.275	0.618
**Right MTG**	A+N-	$PRS1_{noAPOE}$	0.099	0.118	114	0.406	0.649
**Right MTG**	A+N-	$PRS2_{noAPOE}$	0.097	0.118	114	0.413	0.649
**Left STG**	A+N-	$PRS1_{noAPOE}$	0.062	0.064	114	0.332	0.629

**Left STG**	A+N-	$PRS2_{noAPOE}$	0.087	0.065	114	0.179	0.582
**Right STG**	A+N-	$PRS1_{noAPOE}$	0.073	0.088	114	0.408	0.649
**Right STG**	A+N-	$PRS2_{noAPOE}$	0.084	0.089	114	0.352	0.646
**Left FG**	A+N-	$PRS1_{noAPOE}$	0.073	0.055	114	0.192	0.582
**Left FG**	A+N-	$PRS2_{noAPOE}$	0.075	0.055	114	0.182	0.582
**Right FG**	A+N-	$PRS1_{noAPOE}$	0.082	0.055	114	0.139	0.582
**Right FG**	A+N-	$PRS2_{noAPOE}$	0.080	0.055	114	0.150	0.582
**Left mPFC**	A+N-	$PRS1_{noAPOE}$	0.005	0.020	114	0.801	0.866
**Left mPFC**	A+N-	$PRS2_{noAPOE}$	0.004	0.020	114	0.851	0.878
**Right mPFC**	A+N-	$PRS1_{noAPOE}$	0.022	0.026	114	0.409	0.649
**Right mPFC**	A+N-	$PRS2_{noAPOE}$	0.023	0.026	114	0.385	0.649
**Left PCC**	A+N-	$PRS1_{noAPOE}$	0.016	0.016	114	0.312	0.624
**Left PCC**	A+N-	$PRS2_{noAPOE}$	0.019	0.016	114	0.235	0.582
**Right PCC**	A+N-	$PRS1_{noAPOE}$	0.011	0.011	114	0.290	0.618
**Right PCC**	A+N-	$PRS2_{noAPOE}$	0.013	0.011	114	0.210	0.582

FG: Fusiform gyrus; HIP: Hippocampus; mPFC: medial pre-frontal cortex; MTG: middle temporal gyrus; PHG: Parahippocampal gyrus; PCC: posterior cingulate cortex; STG: superior temporal gyrus.

β: standardised regression coefficient for PRS; SE: robust standard error (HC3) of β; N: number of participants included in the model for that row; p: two-sided raw p-value for the PRS coefficient; q: BH-FDR-adjusted p-value (q-value) computed jointly across ROIs, groups, and PRSs (m=96), significance threshold $q < 0.05$.

Table S10. Significant associations between PRSs and regional grey matter volumes within individual groups with concordant CSF and PET biomarkers.

	PRS1			PRS2			$PRS1_{noAPOE}$			$PRS2_{noAPOE}$		
	β	SE	*p*	β	SE	*p*	β	SE	*p*	β	SE	*p*
**A+N+**												
L AMY	n.s.	n.s.	n.s.	-0.021	0.010	0.041	-0.035	0.011	0.001	-0.035	0.011	0.001
L HIP	n.s.	n.s.	n.s.	n.s.	n.s.	n.s.	-0.097	0.033	0.003	-0.098	0.032	0.002
R HIP	n.s.	n.s.	n.s.	-0.060	0.026	0.019	n.s.	n.s.	n.s.	n.s.	n.s.	n.s.
L PHG	n.s.	n.s.	n.s.	n.s.	n.s.	n.s.	-0.095	0.038	0.011	-0.097	0.037	0.010
L MTG	n.s.	n.s.	n.s.	n.s.	n.s.	n.s.	-0.249	0.119	0.036	n.s.	n.s.	n.s.
**A-N-**												
R STG	n.s.	n.s.	n.s.	n.s.	n.s.	n.s.	-0.158	0.066	0.017	-0.170	0.068	0.013
R PCC	-0.048	0.016	0.003	-0.049	0.016	0.003	n.s.	n.s.	n.s.	n.s.	n.s.	n.s.
**A+N-**												
L AMY	-0.026	0.012	0.023	-0.025	0.012	0.037	n.s.	n.s.	n.s.	n.s.	n.s.	n.s.

n.s.: Not significant SE: Standard error; L: Left; R: Right; AMY: Amygdala; FG: Fusiform gyrus; HIP: Hippocampus; mPFC: medial pre-frontal cortex; MTG: middle temporal gyrus; PHG: Parahippocampal gyrus; PCC: posterior cingulate cortex; STG: superior temporal gyrus.

Table S11. Significant associations between PRSs and regional grey matter volumes within groups with concordant CSF and PET biomarkers stratified by APOE genotype.

	**PRS1**			**PRS2**			**PRS1$_{noAPOE}$**			**PR2S$_{noAPOE}$**		
	**β**	**SE**	***p***	**β**	**SE**	***p***	**β**	**SE**	***p***	**β**	**SE**	***p***
**A+N+ non-carrier**												
L AMY	-0.043	0.010	<0.001	-0.041	0.017	0.013	n.s.	n.s.	n.s.	-0.035	0.011	0.001
L PHG	0.180	0.075	0.016	n.s.	n.s.	n.s.	n.s.	n.s.	n.s.	-0.097	0.037	0.010
L STG	n.s.	n.s.	n.s.	n.s.	n.s.	n.s.	n.s.	n.s.	n.s.	-0.149	0.064	0.020
R FG	0.158	0.041	<0.001	0.141	0.037	<0.001	0.099	0.045	0.030	0.099	0.046	0.032
**A+N+ carrier**												
L AMY	n.s.	n.s.	n.s.	n.s.	n.s.	n.s.	-0.048	0.014	0.001	-0.044	0.015	0.003
R AMY	n.s.	n.s.	n.s.	n.s.	n.s.	n.s.	-0.033	0.010	0.002	-0.033	0.010	0.001
L HIP	n.s.	n.s.	n.s.	n.s.	n.s.	n.s.	-0.137	0.043	0.001	-0.130	0.044	0.003
L PHG	n.s.	n.s.	n.s.	n.s.	n.s.	n.s.	-0.152	0.045	0.001	-0.139	0.046	0.003
L MTG	n.s.	n.s.	n.s.	n.s.	n.s.	n.s.	-0.258	0.128	0.043	-0.258	0.125	0.039
R mPFC	n.s.	n.s.	n.s.	n.s.	n.s.	n.s.	0.081	0.025	0.002	0.070	0.027	0.011
**A-N- non-carrier**												
R MTG	n.s.	n.s.	n.s.	n.s.	n.s.	n.s.	-0.196	0.085	0.021	-0.195	0.092	0.035
L STG	n.s.	n.s.	n.s.	-0.283	0.136	0.037	n.s.	n.s.	n.s.	-0.119	0.059	0.044
R STG	-0.325	0.130	0.013	-0.326	0.128	0.011	-0.198	0.062	0.001	-0.193	0.059	0.001
R FG	n.s.	n.s.	n.s.	n.s.	n.s.	n.s.	-0.109	0.048	0.022	-0.112	0.047	0.017
**A+N- carrier**												
R AMY	n.s.	n.s.	n.s.	n.s.	n.s.	n.s.	0.022	0.010	0.024	0.026	0.012	0.035
L MTG	n.s.	n.s.	n.s.	0.649	0.231	0.005	n.s.	n.s.	n.s.	n.s.	n.s.	n.s.
L STG	0.244	0.087	0.005	0.249	0.082	0.002	n.s.	n.s.	n.s.	n.s.	n.s.	n.s.
L FG	n.s.	n.s.	n.s.	0.201	0.090	0.026	n.s.	n.s.	n.s.	n.s.	n.s.	n.s.
R PCC	n.s.	n.s.	n.s.	0.034	0.013	0.008	0.046	0.017	0.007	0.046	0.016	0.003

n.s.: Not significant SE: Standard error; L: Left; R: Right; AMY: Amygdala; FG: Fusiform gyrus; HIP: Hippocampus; mPFC: medial pre-frontal cortex; MTG: middle temporal gyrus; PHG: Parahippocampal gyrus; PCC: posterior cingulate cortex; STG: superior temporal gyrus.

Table S12. Significant associations between PRS1 and regional grey matter volumes in the whole sample with concordant CSF and PET biomarkers. Values are β ± SE and associated p-values from regression models.

	**PRS1**	**Age**	**Sex**	**Education**	**TIV**	**MF**
**L AMY**	**-0.043±0.007, <0.001**	**-0.007±0.001, <0.001**	**0.048±0.021, 0.022**	0.002±0.003, 0.543	**0.000±0.000, <0.001**	**0.045±0.018, 0.010**
**R AMY**	**-0.039±0.007, <0.001**	**-0.007±0.001, <0.001**	**0.053±0.020, 0.008**	-0.001±0.003, 0.824	**0.000±0.000, <0.001**	**0.042±0.017, 0.014**
**L HIP**	**-0.149±0.025, <0.001**	**-0.021±0.004, <0.001**	**0.192±0.072, 0.007**	0.006±0.009, 0.503	**0.002±0.000, <0.001**	**0.163±0.060, 0.007**
**R HIP**	**-0.149±0.026, <0.001**	**-0.022±0.004, <0.001**	**0.257±0.073, <0.001**	0.000±0.009, 0.960	**0.002±0.000, <0.001**	**0.171±0.062, 0.006**
**L PHG**	**-0.159±0.028, <0.001**	**-0.024±0.005, <0.001**	**0.223±0.080, 0.005**	0.005±0.010, 0.593	**0.001±0.000, <0.001**	**0.137±0.067, 0.042**
**R PHG**	**-0.142±0.027, <0.001**	**-0.020±0.004, <0.001**	**0.292±0.077, <0.001**	-0.001±0.010, 0.908	**0.002±0.000, <0.001**	**0.192±0.065, 0.003**
**L MTG**	**-0.606±0.121, <0.001**	**-0.097±0.020, <0.001**	0.447±0.344, 0.194	-0.030±0.044, 0.498	**0.007±0.001, <0.001**	0.430±0.290, 0.139
**R MTG**	**-0.572±0.103, <0.001**	**-0.083±0.017, <0.001**	0.573±0.293, 0.051	0.011±0.038, 0.776	**0.006±0.001, <0.001**	**0.560±0.247, 0.024**
**L STG**	**-0.294±0.062, <0.001**	**-0.064±0.010, <0.001**	0.211±0.175, 0.229	-0.009±0.023, 0.680	**0.003±0.001, <0.001**	0.175±0.148, 0.235
**R STG**	**-0.368±0.075, <0.001**	**-0.074±0.012, <0.001**	**0.421±0.212, 0.047**	0.013±0.027, 0.628	**0.004±0.001, <0.001**	**0.356±0.179, 0.047**
**L FG**	**-0.317±0.064, <0.001**	**-0.050±0.010, <0.001**	0.493±0.181, 0.006	0.007±0.023, 0.755	**0.004±0.001, <0.001**	**0.307±0.152, 0.044**
**R FG**	**-0.225±0.053, <0.001**	**-0.053±0.009, <0.001**	**0.678±0.151, <0.001**	-0.023±0.019, 0.236	**0.005±0.000, <0.001**	**0.475±0.127, <0.001**
**L mPFC**	**-0.056±0.016, 0.001**	**-0.013±0.003, <0.001**	0.046±0.047, 0.328	-0.006±0.006, 0.287	**0.001±0.000, <0.001**	-0.013±0.039,0.733
**R mPFC**	**-0.073±0.020, <0.001**	**-0.015±0.003, <0.001**	0.061±0.058, 0.294	-0.002±0.007, 0.827	**0.001±0.000, <0.001**	0.007±0.049, 0.886
**L PCC**	**-0.064±0.013, <0.001**	**-0.011±0.002, <0.001**	0.071±0.037, 0.057	0.001±0.005, 0.870	**0.001±0.000, <0.001**	0.050±0.032, 0.117
**R PCC**	**-0.040±0.009, <0.001**	**-0.006±0.001, <0.001**	**0.089±0.025, <0.001**	0.001±0.003, 0.769	**0.000±0.000, <0.001**	0.034±0.021, 0.102

L: Left; R: Right; AMY: Amygdala; FG: Fusiform gyrus; HIP: Hippocampus; mPFC: medial pre-frontal cortex; MTG: middle temporal gyrus; PHG: Parahippocampal gyrus; PCC: posterior cingulate cortex; STG: superior temporal gyrus; MF: magnetic field; TIV: total intracranial volume.

The table reports only results for those brain regions where at least one predictor was significantly associated with grey matter volume.

In bold: significant associations between predictors and regional volumes.

Table S13. Significant associations between PRS2 and regional grey matter volumes in the whole sample with concordant CSF and PET biomarkers. Values are β ± SE and associated p-values from regression models.

	PRS2	Age	Sex	Education	TIV	MF
**L AMY**	**-0.043±0.007, <0.001**	**-0.007±0.001, <0.001**	**0.048±0.021, 0.021**	0.002±0.003, 0.560	**0.000±0.000, <0.001**	**0.045±0.018, 0.011**
**R AMY**	**-0.039±0.007, <0.001**	**-0.007±0.001, <0.001**	**0.053±0.020, 0.008**	-0.001±0.003, 0.837	**0.000±0.000, <0.001**	**0.042±0.017, 0.013**
**L HIP**	**-0.147±0.025, <0.001**	**-0.021±0.004, <0.001**	**0.192±0.072, 0.008**	0.006±0.009, 0.506	**0.002±0.000, <0.001**	**0.163±0.061, 0.007**
**R HIP**	**-0.147±0.025, <0.001**	**-0.022±0.004, <0.001**	**0.256±0.072, <0.001**	0.000±0.009, 0.960	**0.002±0.000, <0.001**	**0.171±0.061, 0.005**
**L PHG**	**-0.156±0.028, <0.001**	**-0.024±0.005, <0.001**	**0.222±0.079, 0.005**	0.005±0.010, 0.593	**0.001±0.000, <0.001**	**0.137±0.067, 0.039**
**R PHG**	**-0.140±0.027, <0.001**	**-0.020±0.004, <0.001**	**0.291±0.076, <0.001**	-0.001±0.010, 0.899	**0.002±0.000, <0.001**	**0.192±0.064, 0.003**
**L MTG**	**-0.588±0.120, <0.001**	**-0.097±0.020, <0.001**	0.445±0.341, 0.192	-0.031±0.044, 0.482	**0.007±0.001, <0.001**	0.434±0.288, 0.132
**R MTG**	**-0.558±0.104, <0.001**	**-0.083±0.017, <0.001**	0.573±0.295, 0.052	0.011±0.038, 0.778	**0.006±0.001, <0.001**	**0.561±0.249, 0.024**
**L STG**	**-0.290±0.062, <0.001**	**-0.064±0.010, <0.001**	0.208±0.175, 0.236	-0.010±0.023, 0.670	**0.003±0.001, <0.001**	0.176±0.148, 0.234
**R STG**	**-0.363±0.074, <0.001**	**-0.074±0.012, <0.001**	**0.419±0.211, 0.047**	0.013±0.027, 0.623	**0.004±0.001, <0.001**	**0.358±0.178, 0.044**
**L FG**	**-0.307±0.064, <0.001**	**-0.050±0.010, <0.001**	**0.493±0.183, 0.007**	0.007±0.024, 0.759	**0.004±0.001, <0.001**	**0.307±0.155, 0.048**
**R FG**	**-0.223±0.054, <0.001**	**-0.053±0.009, <0.001**	**0.677±0.153, <0.001**	-0.023±0.020, 0.248	**0.005±0.001, <0.001**	**0.473±0.129, <0.001**
**L mPFC**	**-0.053±0.016, 0.001**	**-0.013±0.003, <0.001**	0.044±0.046, 0.336	-0.006±0.006, 0.276	**0.001±0.000, <0.001**	-0.013±0.039,0.734
**R mPFC**	**-0.070±0.021, 0.001**	**-0.015±0.003, <0.001**	0.059±0.058, 0.312	-0.002±0.008, 0.815	**0.001±0.000, <0.001**	0.007±0.049, 0.888
**L PCC**	**-0.062±0.013, <0.001**	**-0.011±0.002, <0.001**	0.072±0.037, 0.052	0.001±0.005, 0.868	**0.001±0.000, <0.001**	0.050±0.031, 0.111
**R PCC**	**-0.039±0.009, <0.001**	**-0.006±0.001, <0.001**	**0.089±0.025, <0.001**	0.001±0.003, 0.766	**0.000±0.000, <0.001**	0.034±0.021, 0.102

L: Left; R: Right; AMY: Amygdala; FG: Fusiform gyrus; HIP: Hippocampus; mPFC: medial pre-frontal cortex; MTG: middle temporal gyrus; PHG: Parahippocampal gyrus; PCC: posterior cingulate cortex; STG: superior temporal gyrus. MF: magnetic field; TIV: total intracranial volume.

The table reports only results for those brain regions where at least one predictor was significantly associated with grey matter volume.

In bold: significant associations between predictors and regional volumes.

Table S14. Significant associations between PRS1$_{noAPOE}$ and regional grey matter volumes in the whole sample with concordant CSF and PET biomarkers. Values are β ± SE and associated p-values from regression models.

	**PRS1$_{noAPOE}$**	**Age**	**Sex**	**Education**	**TIV**	**MF**
**L AMY**	**-0.022±0.008, 0.005**	**-0.006±0.001, <0.001**	0.038±0.022, 0.089	0.002±0.003, 0.555	**0.000±0.000, <0.001**	**0.044±0.019, 0.019**
**R AMY**	**-0.019±0.007, 0.011**	**-0.006±0.001, <0.001**	**0.044±0.021, 0.039**	-0.001±0.003, 0.821	**0.000±0.000, <0.001**	**0.048±0.018, 0.007**
**L HIP**	**-0.079±0.027, 0.004**	**-0.018±0.004, <0.001**	0.143±0.078, 0.066	0.007±0.010, 0.493	**0.002±0.000, <0.001**	**0.176±0.065, 0.007**
**R HIP**	**-0.064±0.027, 0.017**	**-0.019±0.004, <0.001**	**0.215±0.077, 0.005**	-0.001±0.010, 0.914	**0.002±0.000, <0.001**	**0.185±0.065, 0.004**
**L PHG**	**-0.080±0.029, 0.007**	**-0.021±0.005, <0.001**	**0.169±0.085, 0.045**	0.005±0.011, 0.635	**0.001±0.000, <0.001**	**0.154±0.071, 0.030**
**R PHG**	**-0.054±0.028, 0.052**	**-0.017±0.005, <0.001**	**0.257±0.081, 0.001**	-0.002±0.010, 0.854	**0.002±0.000, <0.001**	**0.210±0.068, 0.002**
**L MTG**	**-0.270±0.123, 0.029**	**-0.087±0.020, <0.001**	0.309±0.356, 0.385	-0.035±0.046, 0.448	**0.007±0.001, <0.001**	0.520±0.298, 0.081
**R MTG**	**-0.245±0.110, 0.025**	**-0.073±0.018, <0.001**	0.361±0.316, 0.254	0.004±0.041, 0.918	**0.007±0.001, <0.001**	**0.632±0.265, 0.017**
**L STG**	**-0.129±0.064, 0.045**	**-0.059±0.011, <0.001**	0.137±0.186, 0.462	-0.011±0.024, 0.633	**0.003±0.001, <0.001**	0.201±0.156, 0.196
**R STG**	-0.152±0.079, 0.055	**-0.069±0.013, <0.001**	0.306±0.228, 0.180	0.011±0.029, 0.713	**0.004±0.001, <0.001**	**0.397±0.191, 0.038**
**L FG**	**-0.147±0.069, 0.033**	**-0.044±0.011, <0.001**	0.402±0.198, 0.043	0.006±0.025, 0.824	**0.004±0.001, <0.001**	**0.334±0.166, 0.045**
**R FG**	**-0.120±0.055, 0.028**	**-0.049±0.009, <0.001**	**0.612±0.158, 0.000**	-0.023±0.020, 0.264	**0.005±0.001, <0.001**	**0.497±0.133, <0.001**
**L mPFC**	-0.026±0.017, 0.115	**-0.012±0.003, <0.001**	0.027±0.048, 0.577	-0.007±0.006, 0.281	**0.001±0.000, <0.001**	-0.007±0.040,0.852
**R mPFC**	-0.019±0.021, 0.356	**-0.014±0.003, <0.001**	0.044±0.060, 0.469	-0.002±0.008, 0.806	**0.001±0.000, <0.001**	0.013±0.050, 0.791
**L PCC**	-0.018±0.014, 0.183	**-0.010±0.002, <0.001**	0.057±0.039, 0.146	0.000±0.005, 0.936	**0.001±0.000, <0.001**	0.060±0.033, 0.067
**R PCC**	-0.013±0.010, 0.189	**-0.005±0.002, 0.001**	**0.076±0.028, 0.007**	0.001±0.004, 0.838	**0.000±0.000, <0.001**	0.039±0.023, 0.095

L: Left; R: Right; AMY: Amygdala; FG: Fusiform gyrus; HIP: Hippocampus; mPFC: medial pre-frontal cortex; MTG: middle temporal gyrus; PHG: Parahippocampal gyrus; PCC: posterior cingulate cortex; STG: superior temporal gyrus; MF: magnetic field; TIV: total intracranial volume.

The table reports only results for those brain regions where at least one predictor was significantly associated with grey matter volume.

In bold: significant associations between predictors and regional volumes.

Table S15. Significant associations between $PRS2_{noAPOE}$ and regional grey matter volumes in the whole sample with concordant CSF and PET biomarkers. Values are β ± SE and associated p-values from regression models.

	**$PRS2_{noAPOE}$**	**Age**	**Sex**	**Education**	**TIV**	**MF**
**L AMY**	**-0.021±0.008, 0.009**	**-0.006±0.001, <0.001**	0.039±0.022, 0.083	0.002±0.003, 0.570	**0.000±0.000, <0.001**	**0.043±0.019, 0.022**
**R AMY**	**-0.017±0.008, 0.029**	**-0.006±0.001, <0.001**	**0.045±0.022, 0.037**	-0.001±0.003, 0.840	**0.000±0.000, <0.001**	**0.047±0.018, 0.009**
**L HIP**	**-0.072±0.027, 0.009**	**-0.018±0.004, <0.001**	0.150±0.078, 0.055	0.007±0.010, 0.498	**0.002±0.000, <0.001**	**0.174±0.065, 0.008**
**R HIP**	**-0.060±0.026, 0.024**	**-0.018±0.004, <0.001**	**0.219±0.075, 0.004**	-0.001±0.010, 0.916	**0.002±0.000, <0.001**	**0.185±0.063, 0.004**
**L PHG**	**-0.073±0.030, 0.017**	**-0.020±0.005, <0.001**	**0.176±0.087, 0.043**	0.005±0.011, 0.646	**0.001±0.000, <0.001**	**0.152±0.073, 0.037**
**R PHG**	-0.050±0.028, 0.078	**-0.017±0.005, <0.001**	**0.260±0.081, 0.001**	-0.002±0.010, 0.849	**0.002±0.000, <0.001**	**0.209±0.068, 0.002**
**L MTG**	-0.239±0.125, 0.056	**-0.086±0.020, <0.001**	0.329±0.356, 0.356	-0.034±0.046, 0.454	**0.007±0.001, <0.001**	0.511±0.299, 0.088
**R MTG**	**-0.222±0.111, 0.046**	**-0.072±0.018, <0.001**	0.363±0.317, 0.252	0.004±0.041, 0.928	**0.006±0.001, <0.001**	**0.627±0.266, 0.018**
**L STG**	-0.114±0.066, 0.084	**-0.059±0.011, <0.001**	0.142±0.187, 0.448	-0.011±0.024, 0.636	**0.003±0.001, <0.001**	0.199±0.157, 0.206
**R STG**	-0.141±0.080, 0.079	**-0.068±0.013, <0.001**	0.315±0.229, 0.170	0.010±0.030, 0.723	**0.004±0.001, <0.001**	**0.394±0.192, 0.041**
**L FG**	-0.130±0.068, 0.057	**-0.044±0.011, <0.001**	**0.414±0.195, 0.034**	0.006±0.025, 0.825	**0.004±0.001, <0.001**	**0.330±0.164, 0.044**
**R FG**	**-0.112±0.055, 0.042**	**-0.049±0.009, <0.001**	**0.619±0.158, 0.000**	-0.022±0.020, 0.270	**0.005±0.001, <0.001**	**0.493±0.132, <0.001**
**L mPFC**	-0.024±0.017, 0.152	**-0.012±0.003, <0.001**	0.028±0.048, 0.563	-0.007±0.006, 0.282	**0.001±0.000, <0.001**	-0.008±0.040,0.835
**R mPFC**	-0.015±0.021, 0.461	**-0.014±0.003, <0.001**	0.045±0.060, 0.449	-0.002±0.008, 0.798	**0.001±0.000, <0.001**	0.013±0.050, 0.794
**L PCC**	-0.016±0.014, 0.247	**-0.010±0.002, <0.001**	0.058±0.039, 0.137	0.000±0.005, 0.928	**0.001±0.000, <0.001**	0.059±0.033, 0.071
**R PCC**	-0.012±0.010, 0.222	**-0.005±0.002, 0.001**	**0.077±0.027, 0.005**	0.001±0.003, 0.831	**0.000±0.000, <0.001**	0.039±0.023, 0.090

L: Left; R: Right; AMY: Amygdala; FG: Fusiform gyrus; HIP: Hippocampus; mPFC: medial pre-frontal cortex; MTG: middle temporal gyrus; PHG: Parahippocampal gyrus; PCC: posterior cingulate cortex; STG: superior temporal gyrus; MF: magnetic field; TIV: total intracranial volume.

The table reports only results for those brain regions where at least one predictor was significantly associated with grey matter volume.

In bold: significant associations between predictors and regional volumes.

Table S16. Significant associations between PRSs and regional grey matter volumes within groups with concordant CSF and PET biomarkers stratified by diagnosis and APOE carrier status.

	PRS1			PRS2			$PRS1_{noAPOE}$			$PRS2_{noAPOE}$		
	β	SE	*p*	β	SE	*p*	β	SE	*p*	β	SE	*p*
**CU non-carrier**												
L AMY	n.s.	n.s.	n.s.	n.s.	n.s.	n.s.	-0.020	0.009	0.036	-0.019	0.009	0.041
R AMY	n.s.	n.s.	n.s.	n.s.	n.s.	n.s.	-0.018	0.009	0.042	n.s.	n.s.	n.s.
L STG	n.s.	n.s.	n.s.	n.s.	n.s.	n.s.	n.s.	n.s.	n.s.	-0.152	0.075	0.044
**CU carrier**												
L AMY	n.s.	n.s.	n.s.	n.s.	n.s.	n.s.	0.019	0.003	<0.001	0.023	0.009	0.011
R AMY	0.015	0.007	0.040	n.s.	n.s.	n.s.	0.045	0.017	0.010	-0.035	0.011	0.001
L HIP	n.s.	n.s.	n.s.	n.s.	n.s.	n.s.	0.342	0.065	<0.001	0.314	0.013	<0.001
R HIP	n.s.	n.s.	n.s.	n.s.	n.s.	n.s.	0.331	0.080	<0.001	0.304	0.047	<0.001
L PHG	n.s.	n.s.	n.s.	n.s.	n.s.	n.s.	0.324	0.076	<0.001	0.299	0.023	<0.001
R PHG	n.s.	n.s.	n.s.	n.s.	n.s.	n.s.	0.375	0.099	<0.001	0.345	0.060	<0.001
L MTG	0.510	0.169	0.003	n.s.	n.s.	n.s.	1.659	0.104	<0.001	1.559	0.085	<0.001
L STG	n.s.	n.s.	n.s.	n.s.	n.s.	n.s.	0.436	0.042	<0.001	0.441	0.061	<0.001
R STG	-0.127	0.026	<0.001	-0.123	0.025	<0.001	n.s.	n.s.	n.s.	-0.149	0.064	0.020
L FG	n.s.	n.s.	n.s.	0.381	0.122	0.002	0.998	0.180	<0.001	0.860	0.248	0.001
R FG	n.s.	n.s.	n.s.	n.s.	n.s.	n.s.	0.827	0.236	<0.001	0.766	0.178	<0.001
R mPFC	n.s.	n.s.	n.s.	n.s.	n.s.	n.s.	n.s.	n.s.	n.s.	0.242	0.106	0.023
L PCC	n.s.	n.s.	n.s.	n.s.	n.s.	n.s.	0.266	0.091	0.003	0.250	0.041	<0.001
R PCC	n.s.	n.s.	n.s.	n.s.	n.s.	n.s.	0.158	0.016	<0.001	0.139	0.014	<0.001
**AD non-carrier**												
L AMY	-0.049	0.022	0.025	-0.050	0.022	0.020	-0.048	0.014	0.001	-0.044	0.015	0.003
**AD carrier**												
L AMY	n.s.	n.s.	n.s.	n.s.	n.s.	n.s.	-0.042	0.018	0.022	-0.038	0.019	0.048
L HIP	n.s.	n.s.	n.s.	n.s.	n.s.	n.s.	-0.161	0.064	0.012	-0.151	0.069	0.028
L PHG	n.s.	n.s.	n.s.	n.s.	n.s.	n.s.	-0.157	0.069	0.022	-0.141	0.070	0.044
R PHG	n.s.	n.s.	n.s.	n.s.	n.s.	n.s.	-0.112	0.049	0.022	-0.109	0.047	0.021
L MTG	n.s.	n.s.	n.s.	n.s.	n.s.	n.s.	-0.630	0.306	0.039	n.s.	n.s.	n.s.
R MTG	n.s.	n.s.	n.s.	n.s.	n.s.	n.s.	-0.537	0.142	<0.001	-0.541	0.141	<0.001
L FG	-0.380	0.179	0.034	-0.394	0.180	0.029	n.s.	n.s.	n.s.	-0.112	0.047	0.017
R FG	n.s.	n.s.	n.s.	n.s.	n.s.	n.s.	-0.229	0.114	0.044	-0.260	0.104	0.013

n.s.: Not significant SE: Standard error; L: Left; R: Right; AMY: Amygdala; FG: Fusiform gyrus; HIP: Hippocampus; mPFC: medial pre-frontal cortex; MTG: middle temporal gyrus; PHG: Parahippocampal gyrus; PCC: posterior cingulate cortex; STG: superior temporal gyrus.

Table S17. Associations between PRSs and regional grey matter volumes within groups with concordant CSF and PET biomarkers with joint FDR (q-values) across ROIs, groups, and PRSs.

	Group	PRS	β	SE	N	p	q
**Left Amygdala**	A+N+	PRS1	-0.019	0.010	74	0.065	0.482
**Left Amygdala**	A+N+	PRS2	-0.021	0.010	74	0.045	0.482
**Left Amygdala**	A+N+	$PRS1_{noAPOE}$	-0.035	0.011	74	0.002	0.145
**Left Amygdala**	A+N+	$PRS2_{noAPOE}$	-0.035	0.011	74	0.002	0.145
**Right Amygdala**	A+N+	PRS1	-0.008	0.010	74	0.413	0.690
**Right Amygdala**	A+N+	PRS2	-0.009	0.009	74	0.366	0.632
**Right Amygdala**	A+N+	$PRS1_{noAPOE}$	-0.018	0.010	74	0.077	0.482
**Right Amygdala**	A+N+	$PRS2_{noAPOE}$	-0.019	0.010	74	0.059	0.482
**Left HIP**	A+N+	PRS1	-0.051	0.032	74	0.110	0.482
**Left HIP**	A+N+	PRS2	-0.056	0.031	74	0.080	0.482
**Left HIP**	A+N+	$PRS1_{noAPOE}$	-0.097	0.033	74	0.005	0.153
**Left HIP**	A+N+	$PRS2_{noAPOE}$	-0.098	0.032	74	0.003	0.145
**Right HIP**	A+N+	PRS1	-0.057	0.026	74	0.033	0.482
**Right HIP**	A+N+	PRS2	-0.060	0.026	74	0.022	0.384
**Right HIP**	A+N+	$PRS1_{noAPOE}$	-0.054	0.033	74	0.103	0.482
**Right HIP**	A+N+	$PRS2_{noAPOE}$	-0.060	0.031	74	0.059	0.482
**Left PHG**	A+N+	PRS1	-0.062	0.035	74	0.080	0.482
**Left PHG**	A+N+	PRS2	-0.066	0.035	74	0.062	0.482
**Left PHG**	A+N+	$PRS1_{noAPOE}$	-0.095	0.038	74	0.014	0.314
**Left PHG**	A+N+	$PRS2_{noAPOE}$	-0.097	0.037	74	0.012	0.314
**Right PHG**	A+N+	PRS1	-0.033	0.029	74	0.258	0.572
**Right PHG**	A+N+	PRS2	-0.036	0.029	74	0.224	0.558
**Right PHG**	A+N+	$PRS1_{noAPOE}$	-0.042	0.034	74	0.222	0.558
**Right PHG**	A+N+	$PRS2_{noAPOE}$	-0.053	0.035	74	0.130	0.482
**Left MTG**	A+N+	PRS1	-0.114	0.118	74	0.336	0.608

**Left MTG**	A+N+	PRS2	-0.117	0.116	74	0.315	0.606
**Left MTG**	A+N+	$PRS1_{noAPOE}$	-0.249	0.119	74	0.040	0.482
**Left MTG**	A+N+	$PRS2_{noAPOE}$	-0.234	0.124	74	0.064	0.482
**Right MTG**	A+N+	PRS1	-0.015	0.125	74	0.907	0.966
**Right MTG**	A+N+	PRS2	-0.014	0.124	74	0.913	0.966
**Right MTG**	A+N+	$PRS1_{noAPOE}$	-0.071	0.133	74	0.596	0.753
**Right MTG**	A+N+	$PRS2_{noAPOE}$	-0.084	0.136	74	0.537	0.721
**Left STG**	A+N+	PRS1	-0.033	0.058	74	0.568	0.741
**Left STG**	A+N+	PRS2	-0.032	0.058	74	0.576	0.747
**Left STG**	A+N+	$PRS1_{noAPOE}$	-0.107	0.065	74	0.103	0.482
**Left STG**	A+N+	$PRS2_{noAPOE}$	-0.091	0.067	74	0.179	0.544
**Right STG**	A+N+	PRS1	0.048	0.082	74	0.559	0.735
**Right STG**	A+N+	PRS2	0.049	0.081	74	0.550	0.731
**Right STG**	A+N+	$PRS1_{noAPOE}$	-0.032	0.097	74	0.744	0.861
**Right STG**	A+N+	$PRS2_{noAPOE}$	-0.041	0.096	74	0.667	0.800
**Left FG**	A+N+	PRS1	0.022	0.064	74	0.728	0.852
**Left FG**	A+N+	PRS2	0.016	0.065	74	0.803	0.907
**Left FG**	A+N+	$PRS1_{noAPOE}$	-0.056	0.074	74	0.452	0.692
**Left FG**	A+N+	$PRS2_{noAPOE}$	-0.056	0.073	74	0.449	0.692
**Right FG**	A+N+	PRS1	0.085	0.062	74	0.173	0.544
**Right FG**	A+N+	PRS2	0.078	0.062	74	0.209	0.558
**Right FG**	A+N+	$PRS1_{noAPOE}$	-0.021	0.074	74	0.773	0.883
**Right FG**	A+N+	$PRS2_{noAPOE}$	-0.055	0.075	74	0.470	0.692
**Left mPFC**	A+N+	PRS1	0.019	0.019	74	0.316	0.606
**Left mPFC**	A+N+	PRS2	0.021	0.019	74	0.275	0.591
**Left mPFC**	A+N+	$PRS1_{noAPOE}$	0.014	0.022	74	0.527	0.713
**Left mPFC**	A+N+	$PRS2_{noAPOE}$	0.015	0.022	74	0.486	0.692
**Right mPFC**	A+N+	PRS1	0.028	0.023	74	0.235	0.558

**Right mPFC**	A+N+	PRS2	0.030	0.024	74	0.223	0.558
**Right mPFC**	A+N+	$PRS1_{noAPOE}$	0.044	0.026	74	0.100	0.482
**Right mPFC**	A+N+	$PRS2_{noAPOE}$	0.039	0.026	74	0.139	0.482
**Left PCC**	A+N+	PRS1	-0.029	0.016	74	0.075	0.482
**Left PCC**	A+N+	PRS2	-0.028	0.016	74	0.076	0.482
**Left PCC**	A+N+	$PRS1_{noAPOE}$	0.023	0.022	74	0.295	0.593
**Left PCC**	A+N+	$PRS2_{noAPOE}$	0.020	0.021	74	0.351	0.623
**Right PCC**	A+N+	PRS1	-0.009	0.009	74	0.359	0.627
**Right PCC**	A+N+	PRS2	-0.008	0.009	74	0.387	0.657
**Right PCC**	A+N+	$PRS1_{noAPOE}$	0.002	0.011	74	0.853	0.939
**Right PCC**	A+N+	$PRS2_{noAPOE}$	0.003	0.011	74	0.820	0.916
**Left Amygdala**	A-N-	PRS1	-0.010	0.011	88	0.387	0.657
**Left Amygdala**	A-N-	PRS2	-0.011	0.011	88	0.344	0.618
**Left Amygdala**	A-N-	$PRS1_{noAPOE}$	-0.009	0.007	88	0.187	0.544
**Left Amygdala**	A-N-	$PRS2_{noAPOE}$	-0.008	0.007	88	0.231	0.558
**Right Amygdala**	A-N-	PRS1	-0.012	0.010	88	0.238	0.558
**Right Amygdala**	A-N-	PRS2	-0.012	0.010	88	0.244	0.558
**Right Amygdala**	A-N-	$PRS1_{noAPOE}$	-0.007	0.007	88	0.333	0.608
**Right Amygdala**	A-N-	$PRS2_{noAPOE}$	-0.006	0.007	88	0.437	0.692
**Left HIP**	A-N-	PRS1	0.001	0.038	88	0.979	0.990
**Left HIP**	A-N-	PRS2	0.000	0.037	88	0.999	0.999
**Left HIP**	A-N-	$PRS1_{noAPOE}$	-0.019	0.025	88	0.448	0.692
**Left HIP**	A-N-	$PRS2_{noAPOE}$	-0.019	0.025	88	0.449	0.692
**Right HIP**	A-N-	PRS1	0.016	0.036	88	0.661	0.800
**Right HIP**	A-N-	PRS2	0.013	0.035	88	0.708	0.839
**Right HIP**	A-N-	$PRS1_{noAPOE}$	-0.009	0.022	88	0.701	0.836
**Right HIP**	A-N-	$PRS2_{noAPOE}$	-0.012	0.023	88	0.594	0.753
**Left PHG**	A-N-	PRS1	0.004	0.041	88	0.926	0.966

**Left PHG**	A-N-	PRS2	0.004	0.040	88	0.928	0.966
**Left PHG**	A-N-	$PRS1_{noAPOE}$	-0.019	0.027	88	0.486	0.692
**Left PHG**	A-N-	$PRS2_{noAPOE}$	-0.018	0.027	88	0.505	0.702
**Right PHG**	A-N-	PRS1	0.029	0.041	88	0.483	0.692
**Right PHG**	A-N-	PRS2	0.029	0.041	88	0.476	0.692
**Right PHG**	A-N-	$PRS1_{noAPOE}$	0.001	0.027	88	0.958	0.974
**Right PHG**	A-N-	$PRS2_{noAPOE}$	0.000	0.027	88	0.989	0.994
**Left MTG**	A-N-	PRS1	-0.127	0.180	88	0.482	0.692
**Left MTG**	A-N-	PRS2	-0.132	0.178	88	0.460	0.692
**Left MTG**	A-N-	$PRS1_{noAPOE}$	-0.120	0.114	88	0.296	0.593
**Left MTG**	A-N-	$PRS2_{noAPOE}$	-0.138	0.117	88	0.243	0.558
**Right MTG**	A-N-	PRS1	-0.187	0.158	88	0.239	0.558
**Right MTG**	A-N-	PRS2	-0.188	0.156	88	0.232	0.558
**Right MTG**	A-N-	$PRS1_{noAPOE}$	-0.156	0.095	88	0.106	0.482
**Right MTG**	A-N-	$PRS2_{noAPOE}$	-0.170	0.097	88	0.085	0.482
**Left STG**	A-N-	PRS1	-0.157	0.091	88	0.088	0.482
**Left STG**	A-N-	PRS2	-0.165	0.089	88	0.069	0.482
**Left STG**	A-N-	$PRS1_{noAPOE}$	-0.101	0.059	88	0.092	0.482
**Left STG**	A-N-	$PRS2_{noAPOE}$	-0.113	0.061	88	0.069	0.482
**Right STG**	A-N-	PRS1	-0.184	0.113	88	0.107	0.482
**Right STG**	A-N-	PRS2	-0.189	0.113	88	0.099	0.482
**Right STG**	A-N-	$PRS1_{noAPOE}$	-0.158	0.066	88	0.019	0.370
**Right STG**	A-N-	$PRS2_{noAPOE}$	-0.170	0.068	88	0.015	0.314
**Left FG**	A-N-	PRS1	-0.066	0.104	88	0.527	0.713
**Left FG**	A-N-	PRS2	-0.067	0.104	88	0.519	0.711
**Left FG**	A-N-	$PRS1_{noAPOE}$	-0.075	0.063	88	0.240	0.558
**Left FG**	A-N-	$PRS2_{noAPOE}$	-0.082	0.065	88	0.210	0.558
**Right FG**	A-N-	PRS1	-0.043	0.089	88	0.632	0.783

**Right FG**	A-N-	PRS2	-0.040	0.088	88	0.650	0.796
**Right FG**	A-N-	$PRS1_{noAPOE}$	-0.086	0.058	88	0.140	0.482
**Right FG**	A-N-	$PRS2_{noAPOE}$	-0.085	0.058	88	0.149	0.498
**Left mPFC**	A-N-	PRS1	-0.003	0.026	88	0.902	0.966
**Left mPFC**	A-N-	PRS2	-0.005	0.026	88	0.856	0.939
**Left mPFC**	A-N-	$PRS1_{noAPOE}$	-0.025	0.016	88	0.112	0.482
**Left mPFC**	A-N-	$PRS2_{noAPOE}$	-0.029	0.016	88	0.082	0.482
**Right mPFC**	A-N-	PRS1	-0.019	0.028	88	0.515	0.711
**Right mPFC**	A-N-	PRS2	-0.020	0.028	88	0.484	0.692
**Right mPFC**	A-N-	$PRS1_{noAPOE}$	-0.026	0.017	88	0.141	0.482
**Right mPFC**	A-N-	$PRS2_{noAPOE}$	-0.027	0.018	88	0.138	0.482
**Left PCC**	A-N-	PRS1	-0.032	0.021	88	0.137	0.482
**Left PCC**	A-N-	PRS2	-0.032	0.020	88	0.122	0.482
**Left PCC**	A-N-	$PRS1_{noAPOE}$	-0.021	0.014	88	0.126	0.482
**Left PCC**	A-N-	$PRS2_{noAPOE}$	-0.023	0.014	88	0.095	0.482
**Right PCC**	A-N-	PRS1	-0.048	0.016	88	0.004	0.145
**Right PCC**	A-N-	PRS2	-0.049	0.016	88	0.003	0.145
**Right PCC**	A-N-	$PRS1_{noAPOE}$	-0.011	0.011	88	0.329	0.608
**Right PCC**	A-N-	$PRS2_{noAPOE}$	-0.012	0.011	88	0.278	0.591
**Left Amygdala**	A+N-	PRS1	-0.026	0.012	56	0.027	0.439
**Left Amygdala**	A+N-	PRS2	-0.025	0.012	56	0.043	0.482
**Left Amygdala**	A+N-	$PRS1_{noAPOE}$	-0.003	0.013	56	0.818	0.916
**Left Amygdala**	A+N-	$PRS2_{noAPOE}$	-0.001	0.013	56	0.932	0.966
**Right Amygdala**	A+N-	PRS1	-0.019	0.011	56	0.097	0.482
**Right Amygdala**	A+N-	PRS2	-0.017	0.011	56	0.119	0.482
**Right Amygdala**	A+N-	$PRS1_{noAPOE}$	0.003	0.011	56	0.756	0.869
**Right Amygdala**	A+N-	$PRS2_{noAPOE}$	0.007	0.011	56	0.552	0.731
**Left HIP**	A+N-	PRS1	-0.054	0.030	56	0.082	0.482

**Left HIP**	A+N-	PRS2	-0.051	0.030	56	0.097	0.482
**Left HIP**	A+N-	$PRS1_{noAPOE}$	-0.015	0.034	56	0.665	0.800
**Left HIP**	A+N-	$PRS2_{noAPOE}$	-0.007	0.034	56	0.840	0.932
**Right HIP**	A+N-	PRS1	-0.059	0.037	56	0.118	0.482
**Right HIP**	A+N-	PRS2	-0.059	0.037	56	0.120	0.482
**Right HIP**	A+N-	$PRS1_{noAPOE}$	0.004	0.040	56	0.926	0.966
**Right HIP**	A+N-	$PRS2_{noAPOE}$	0.012	0.041	56	0.781	0.887
**Left PHG**	A+N-	PRS1	-0.050	0.035	56	0.162	0.526
**Left PHG**	A+N-	PRS2	-0.048	0.035	56	0.178	0.544
**Left PHG**	A+N-	$PRS1_{noAPOE}$	-0.005	0.038	56	0.896	0.966
**Left PHG**	A+N-	$PRS2_{noAPOE}$	0.003	0.038	56	0.936	0.966
**Right PHG**	A+N-	PRS1	-0.048	0.037	56	0.198	0.558
**Right PHG**	A+N-	PRS2	-0.047	0.036	56	0.200	0.558
**Right PHG**	A+N-	$PRS1_{noAPOE}$	0.022	0.041	56	0.600	0.753
**Right PHG**	A+N-	$PRS2_{noAPOE}$	0.031	0.041	56	0.457	0.692
**Left MTG**	A+N-	PRS1	0.123	0.169	56	0.469	0.692
**Left MTG**	A+N-	PRS2	0.138	0.170	56	0.420	0.692
**Left MTG**	A+N-	$PRS1_{noAPOE}$	0.087	0.168	56	0.605	0.755
**Left MTG**	A+N-	$PRS2_{noAPOE}$	0.095	0.171	56	0.580	0.747
**Right MTG**	A+N-	PRS1	-0.210	0.138	56	0.134	0.482
**Right MTG**	A+N-	PRS2	-0.202	0.138	56	0.151	0.498
**Right MTG**	A+N-	$PRS1_{noAPOE}$	0.047	0.141	56	0.739	0.860
**Right MTG**	A+N-	$PRS2_{noAPOE}$	0.052	0.145	56	0.724	0.852
**Left STG**	A+N-	PRS1	0.056	0.080	56	0.483	0.692
**Left STG**	A+N-	PRS2	0.060	0.079	56	0.450	0.692
**Left STG**	A+N-	$PRS1_{noAPOE}$	0.094	0.082	56	0.259	0.572
**Left STG**	A+N-	$PRS2_{noAPOE}$	0.106	0.079	56	0.183	0.544
**Right STG**	A+N-	PRS1	-0.118	0.108	56	0.280	0.591

**Right STG**	A+N-	PRS2	-0.117	0.109	56	0.291	0.593
**Right STG**	A+N-	$PRS1_{noAPOE}$	0.062	0.118	56	0.600	0.753
**Right STG**	A+N-	$PRS2_{noAPOE}$	0.054	0.119	56	0.651	0.796
**Left FG**	A+N-	PRS1	-0.006	0.067	56	0.929	0.966
**Left FG**	A+N-	PRS2	-0.004	0.067	56	0.956	0.974
**Left FG**	A+N-	$PRS1_{noAPOE}$	0.075	0.070	56	0.292	0.593
**Left FG**	A+N-	$PRS2_{noAPOE}$	0.074	0.070	56	0.294	0.593
**Right FG**	A+N-	PRS1	-0.041	0.059	56	0.497	0.696
**Right FG**	A+N-	PRS2	-0.042	0.061	56	0.497	0.696
**Right FG**	A+N-	$PRS1_{noAPOE}$	0.074	0.055	56	0.184	0.544
**Right FG**	A+N-	$PRS2_{noAPOE}$	0.074	0.055	56	0.182	0.544
**Left mPFC**	A+N-	PRS1	0.024	0.023	56	0.311	0.606
**Left mPFC**	A+N-	PRS2	0.024	0.023	56	0.303	0.600
**Left mPFC**	A+N-	$PRS1_{noAPOE}$	0.026	0.023	56	0.270	0.589
**Left mPFC**	A+N-	$PRS2_{noAPOE}$	0.026	0.023	56	0.258	0.572
**Right mPFC**	A+N-	PRS1	0.023	0.031	56	0.458	0.692
**Right mPFC**	A+N-	PRS2	0.023	0.031	56	0.461	0.692
**Right mPFC**	A+N-	$PRS1_{noAPOE}$	0.031	0.034	56	0.357	0.627
**Right mPFC**	A+N-	$PRS2_{noAPOE}$	0.030	0.035	56	0.397	0.669
**Left PCC**	A+N-	PRS1	-0.002	0.021	56	0.927	0.966
**Left PCC**	A+N-	PRS2	-0.001	0.021	56	0.956	0.974
**Left PCC**	A+N-	$PRS1_{noAPOE}$	0.020	0.021	56	0.334	0.608
**Left PCC**	A+N-	$PRS2_{noAPOE}$	0.020	0.021	56	0.331	0.608
**Right PCC**	A+N-	PRS1	0.017	0.014	56	0.227	0.558
**Right PCC**	A+N-	PRS2	0.017	0.014	56	0.238	0.558
**Right PCC**	A+N-	$PRS1_{noAPOE}$	0.018	0.015	56	0.244	0.558
**Right PCC**	A+N-	$PRS2_{noAPOE}$	0.015	0.016	56	0.330	0.608

FG: Fusiform gyrus; HIP: Hippocampus; mPFC: medial pre-frontal cortex; MTG: middle temporal gyrus; PHG: Parahippocampal gyrus; PCC: posterior cingulate cortex; STG: superior temporal gyrus.

β: standardised regression coefficient for PRS; SE: robust standard error (HC3) of β; N: number of participants included in the model for that row; p: two-sided raw p-value for the PRS coefficient; q: BH-FDR-adjusted p-value (q-value) computed jointly across ROIs, groups, and PRSs (m=96), significance threshold $q < 0.05$.

Statistical analysis - R scripts

```
# Comparison of PRSs across groups #

kw_PRSx <- kruskal.test(PRSx ~ categorizzazione, data = df)
dunn.test::dunn.test(df$PRSx, df$categorizzazione, method = "bonferroni", kw = TRUE, list = TRUE)

# Associations between PRSs and regional GM volumes within individual groups #

# Analysis: within each "categorizzazione" group
# Robust regression (MASS::rlm)
# Model: GM_region ~ PRSx + AGE + sesso + EDU + PC1 + PC2 + PC3 + PC4 + PC5 + TIV + MRF_strength
# Extract Beta, SE, t-value and p-value (calculated from the normal distribution)

# Define GM regions
gm_vars <- c("AmygdalaL","AmygdalaR","HippocampusL","HippocampusR",
             "ParahippocampalGyrusL","ParahippocampalGyrusR",
             "MiddleTemporalGyrusL","MiddleTemporalGyrusR",
             "SuperiorTemporalGyrusL","SuperiorTemporalGyrusR",
             "FusiformGyrusL","FusiformGyrusR",
             "MedialPreFrontalCortexL","MedialPreFrontalCortexR",
             "PosteriorCingulateCortexL","PosteriorCingulateCortexR")

# Create empty list for results
results_PRSx <- list()

# Loop over "categorizzazione" groups
for(gr in unique(df$categorizzazione)){

  # 1. Subset dataset for group
  df_sub <- subset(df, categorizzazione == gr)

  # 2. Loop over brain regions
  for(region in gm_vars){

    # Build model formula with TIV and MRF_strength as covariates
    f <- as.formula(paste(region, "~ PRSx + AGE + sesso + EDU + PC1 + PC2 + PC3 + PC4 + PC5 + TIV +
MRF_strength"))

    # Run robust regression
    model <- MASS::rlm(f, data = df_sub)
```

```
    # Extract coefficients
    coef_summary <- summary(model)$coef

    # Check if PRS1 is in the model
    if("PRSx" %in% rownames(coef_summary)){
      prs_coef <- coef_summary["PRS1", ]

      # Compute p-value from t-value using normal distribution
      t_val <- prs_coef[3]
      p_val <- 2 * pnorm(-abs(t_val))

      # Save results into a table
      results_PRSx[[paste(gr, region, sep = "_")]] <- data.frame(
        Group   = gr,
        Region  = region,
        Beta    = prs_coef[1],
        SE      = prs_coef[2],
        t_value = t_val,
        p_value = p_val
      )
    }
  }
}

# Associations between PRSs and regional GM volumes within groups stratified by APOE genotype #

library(MASS)

# Analysis: within each group (categorizzazione × APOE_car)
# Robust regression (MASS::rlm)
# Model: GM_region ~ PRSx + AGE + sesso + EDU + PC1 + PC2 + PC3 + PC4 + PC5 + TIV + MRF_strength
# Extract Beta, SE, t-value and p-value (from the normal distribution)

# Define GM regions
gm_vars <- c("AmygdalaL","AmygdalaR","HippocampusL","HippocampusR",
             "ParahippocampalGyrusL","ParahippocampalGyrusR",
             "MiddleTemporalGyrusL","MiddleTemporalGyrusR",
             "SuperiorTemporalGyrusL","SuperiorTemporalGyrusR",
             "FusiformGyrusL","FusiformGyrusR",
             "MedialPreFrontalCortexL","MedialPreFrontalCortexR",
```

```
            "PosteriorCingulateCortexL","PosteriorCingulateCortexR")

# List for results
results_PRSx_APOE <- list()

# Loop over "categorizzazione" groups
for(gr in unique(df$categorizzazione)){

  df_cat <- subset(df, categorizzazione == gr)

  # Loop over APOE_car (1 = non-carrier, 2 = carrier)
  for(ap in unique(df_cat$APOE_car)){

    df_sub <- subset(df_cat, APOE_car == ap)

    # Loop over regions
    for(region in gm_vars){

      # Model formula with TIV and MRF_strength
      f <- as.formula(paste(region, "~ PRSx + AGE + sesso + EDU + PC1 + PC2 + PC3 + PC4 + PC5 + TIV +
MRF_strength"))

      # Robust regression
      model <- MASS::rlm(f, data = df_sub)

      # Extract coefficients
      coef_summary <- summary(model)$coef

      if("PRSx" %in% rownames(coef_summary)){
        prs_coef <- coef_summary["PRSx", ]

        # Compute p-value from normal distribution
        t_val <- prs_coef[3]
        p_val <- 2 * pnorm(-abs(t_val))

        # Save results
        results_PRSx_APOE[[paste(gr, ap, region, sep = "_")]] <- data.frame(
          Group      = gr,
          APOE_status = ifelse(ap == 1, "Non-carrier", "Carrier"),
          Region      = region,
          Beta        = prs_coef[1],
```

```
          SE          = prs_coef[2],
          t_value     = t_val,
          p_value     = p_val
        )
      }
    }
  }
}

# Combine into one dataframe
results_PRSx_APOE <- do.call(rbind, results_PRSx_APOE)

# Print final table
print(results_PRSx_APOE)

# Associations between PRSs and regional GM volumes in the whole sample #

library(MASS)

# Define GM regions
gm_vars <- c("AmygdalaL","AmygdalaR","HippocampusL","HippocampusR",
             "ParahippocampalGyrusL","ParahippocampalGyrusR",
             "MiddleTemporalGyrusL","MiddleTemporalGyrusR",
             "SuperiorTemporalGyrusL","SuperiorTemporalGyrusR",
             "FusiformGyrusL","FusiformGyrusR",
             "MedialPreFrontalCortexL","MedialPreFrontalCortexR",
             "PosteriorCingulateCortexL","PosteriorCingulateCortexR")

# List for results
results_all <- list()

# Loop over regions
for(region in gm_vars){

  # Model formula with TIV and MRF_strength as covariates
  f <- as.formula(paste(region, "~ PRSx + AGE + sesso + EDU + PC1 + PC2 + PC3 + PC4 + PC5 + TIV +
MRF_strength"))

  # Run robust regression
  model <- MASS::rlm(f, data = df)
  coef_summary <- summary(model)$coef
```

```
  # Extract all predictors
  for (pred in rownames(coef_summary)) {
    beta <- coef_summary[pred, 1]
    se   <- coef_summary[pred, 2]
    tval <- coef_summary[pred, 3]
    pval <- 2 * pnorm(-abs(tval))

    results_all[[paste(region, pred, sep = "_")]] <- data.frame(
      Region    = region,
      Predictor = pred,
      Beta      = round(beta, 3),
      SE        = round(se, 3),
      t_value   = round(tval, 3),
      p_value   = signif(pval, 3)
    )
  }
}

# Combine into one dataframe
results_all <- do.call(rbind, results_all)

# Print full table
print(results_all)

# Associations between PRSs and regional GM volumes stratified by diagnosis and APOE carrier status #

library(MASS)

# Analysis: stratification by clinical diagnosis (Diagnosis) and APOE_car (1 = non-carrier, 2 = carrier)
# Robust regression (MASS::rlm)
# Model: GM_region ~ PRSx + AGE + sesso + EDU + TIV + MRF_strength
# Extract Beta, SE, t-value and p-value (calculated from the normal distribution)

# Define GM regions
gm_vars <- c("AmygdalaL","AmygdalaR","HippocampusL","HippocampusR",
             "ParahippocampalGyrusL","ParahippocampalGyrusR",
             "MiddleTemporalGyrusL","MiddleTemporalGyrusR",
             "SuperiorTemporalGyrusL","SuperiorTemporalGyrusR",
             "FusiformGyrusL","FusiformGyrusR",
             "MedialPreFrontalCortexL","MedialPreFrontalCortexR",
```

```
             "PosteriorCingulateCortexL","PosteriorCingulateCortexR")

results_PRSx_diagAPOE <- list()

# Loop over clinical diagnoses
for(diag in unique(df$Diagnosis)){

  df_diag <- subset(df, Diagnosis == diag)

  # Loop over APOE_car
  for(ap in unique(df_diag$APOE_car)){

    df_sub <- subset(df_diag, APOE_car == ap)

    # Loop over GM regions
    for(region in gm_vars){

      # Model formula with TIV and MRF_strength
      f <- as.formula(paste(region, "~ PRSx + AGE + sesso + EDU + TIV + MRF_strength"))

      # Robust regression
      model <- MASS::rlm(f, data = df_sub)

      # Extract coefficients
      coef_summary <- summary(model)$coef

      if("PRSx" %in% rownames(coef_summary)){
        prs_coef <- coef_summary["PRS1", ]

        # Compute p-value from normal distribution
        t_val <- prs_coef[3]
        p_val <- 2 * pnorm(-abs(t_val))

        # Save results
        results_PRSx_diagAPOE[[paste(diag, ap, region, sep = "_")]] <- data.frame(
          Diagnosis   = diag,
          APOE_status = ifelse(ap == 1, "Non-carrier", "Carrier"),
          Region      = region,
          Beta        = prs_coef[1],
          SE          = prs_coef[2],
          t_value     = t_val,
```

```
        p_value     = p_val
      )
    }
  }
 }
}
```

ROI-wise associations between PRSs and regional grey-matter volume with joint FDR across ROIs, groups, and PRSs

```
# Analysis: ROI-wise associations between PRSs and regional GM volumes
# Stratification: categorizzazione (A+N+, A+N-, A-N-)
# Model: robust regression (MASS::rlm)
# Covariates: AGE, SEX, EDU, PC1, PC2, PC3, PC4, PC5, TIV, MRF_strength
# Number of tests: 16 ROI × 3 groups × 2 PRS = 96
# Output: Beta, SE, N, p (Student's t), q (BH-FDR on m=96)

library(MASS)

# 1. Define ROIs
gm_vars <- c("AmygdalaL","AmygdalaR","HippocampusL","HippocampusR",
             "ParahippocampalGyrusL","ParahippocampalGyrusR",
             "MiddleTemporalGyrusL","MiddleTemporalGyrusR",
             "SuperiorTemporalGyrusL","SuperiorTemporalGyrusR",
             "FusiformGyrusL","FusiformGyrusR",
             "MedialPreFrontalCortexL","MedialPreFrontalCortexR",
             "PosteriorCingulateCortexL","PosteriorCingulateCortexR")

# 2. List for results
all_results <- list()

# 3. Function for robust regression
run_rlm <- function(df_sub, formula, prs_name, group, region){
  model <- MASS::rlm(formula, data = df_sub)
  coef_summary <- summary(model)$coef
  if(prs_name %in% rownames(coef_summary)){
    prs_coef <- coef_summary[prs_name, ]
    t_val <- prs_coef[3]
    # df = N - k
    df_resid <- nrow(df_sub) - length(coef(model))
    # p-value from t distribution
```

```
    p_val <- 2 * pt(-abs(t_val), df = df_resid)
    return(data.frame(
      Group   = group,
      PRS     = prs_name,
      Region  = region,
      Beta    = prs_coef[1],
      SE      = prs_coef[2],
      N       = nrow(df_sub),
      p_value = p_val
    ))
  } else {
    return(NULL)
  }
}

# 4. Loop over groups, ROIs and PRS
for(gr in unique(df$categorizzazione)){
  df_sub <- subset(df, categorizzazione == gr)
  for(region in gm_vars){
    for(prs in c("PRS1","PRS2")){
      f <- as.formula(paste(region, "~", prs,
                            "+ AGE + sesso + EDU + PC1 + PC2 + PC3 + PC4 + PC5 + TIV + MRF_strength"))
      res <- run_rlm(df_sub, f, prs, gr, region)
      if(!is.null(res)) all_results[[length(all_results)+1]] <- res
    }
  }
}

# 5. Combine into single dataframe
all_results <- do.call(rbind, all_results)

# 6. FDR correction across m = 96 tests
all_results$q_value <- p.adjust(all_results$p_value, method = "BH", n = 96)

# 7. Check total number of rows
print(nrow(all_results))  # should return 96

# 8. View results
print(all_results)
```

The same pipeline was used for $PRS1_{noAPOE}$ and $PRS2_{noAPOE}$.

All the analysis on concord subjects used the same scripts, but on the df_concord1 dataframe generated as follows:

```
# Subset of subjects with Conc..1.yes..2.no. == 1
df_concord1 <- subset(df, df$`Conc..1.yes..2.no.` == 1)
```

For the associations between PRSs and regional GM volumes within groups stratified by APOE genotype, we replaced MASS rlm with robustbase lmrob because, in some subsets of the data, rlm failed to converge or produced singular fits due to collinearity among covariates or small sample sizes. The function lmrob implements more advanced robust estimation methods (MM-estimators with S-estimates for high breakdown point and efficiency), which are more stable under multicollinearity and heteroskedasticity. Therefore, it provides reliable estimates in contexts where rlm may fail. In addition, we wrapped the model fitting step inside a tryCatch block to handle potential convergence failures gracefully. This ensures that the analysis continues even if a specific subgroup does not provide enough variability to estimate all coefficients reliably.

Example: R SCRIPT FOR PRS1$_{noAPOE}$

```
library(robustbase)

# GM regions
gm_vars <- c("AmygdalaL","AmygdalaR","HippocampusL","HippocampusR",
             "ParahippocampalGyrusL","ParahippocampalGyrusR",
             "MiddleTemporalGyrusL","MiddleTemporalGyrusR",
             "SuperiorTemporalGyrusL","SuperiorTemporalGyrusR",
             "FusiformGyrusL","FusiformGyrusR",
             "MedialPreFrontalCortexL","MedialPreFrontalCortexR",
             "PosteriorCingulateCortexL","PosteriorCingulateCortexR")

results_PRSnoAPOE1_APOE_concord1 <- list()

for(gr in unique(df_concord1$categorizzazione)){
  df_cat <- subset(df_concord1, categorizzazione == gr)

  for(ap in unique(df_cat$APOE_car)){
    df_sub <- subset(df_cat, APOE_car == ap)

    for(region in gm_vars){
      f <- as.formula(paste(region, "~ PRSnoAPOE1 + AGE + sesso + EDU + PC1 + PC2 + PC3 + PC4 + PC5 + TIV
+ MRF_strength"))
```

```
      model <- tryCatch(
        suppressWarnings(lmrob(f, data = df_sub)),
        error = function(e) NULL
      )

      if(!is.null(model)){
        coef_summary <- tryCatch(summary(model)$coef, error = function(e) NULL)

        if(!is.null(coef_summary) && "PRSnoAPOE1" %in% rownames(coef_summary)){
          beta <- coef_summary["PRSnoAPOE1", 1]
          se   <- coef_summary["PRSnoAPOE1", 2]
          tval <- coef_summary["PRSnoAPOE1", 3]
          pval <- 2 * pnorm(-abs(tval))

          results_PRSnoAPOE1_APOE_concord1[[paste(gr, ap, region, sep = "_")]] <- data.frame(
            Group       = gr,
            APOE_status = ifelse(ap == 1, "Non-carrier", "Carrier"),
            Region      = region,
            Beta        = beta,
            SE          = se,
            t_value     = tval,
            p_value     = pval
          )
        }
      }
    }
  }
}

# Combine
if(length(results_PRSnoAPOE1_APOE_concord1) > 0){
  results_PRSnoAPOE1_APOE_concord1 <- do.call(rbind, results_PRSnoAPOE1_APOE_concord1)
  print(results_PRSnoAPOE1_APOE_concord1)
} else {
  message("No valid results for PRSnoAPOE1 in this subset.")
}
```

PRS calculation steps – Linux scripts

```
# Quality check of the ADNI genotypes with PLINK

plink1.9 --bfile WGS_Omni25_BIN_wo_ConsentsIssues --hwe 1e-6 midp --geno 0.1 --maf-succ --maf 0.05 --output-chr 26 --make-bed --out WGS_Omni25_BIN_wo_ConsentsIssues.qc

# Quality check for PRSs without APOE

plink1.9 --bfile WGS_Omni25_BIN_wo_ConsentsIssues --chr 19 --from-bp 44400000 --to-bp 46500000 --write-snplist

plink1.9 --bfile WGS_Omni25_BIN_wo_ConsentsIssues --hwe 1e-6 midp --geno 0.1 --maf-succ --maf 0.05 --exclude plink.snplist --output-chr 26 --make-bed --out WGS_Omni25_BIN_wo_ConsentsIssues.qc

# Summary statistics file filtering (https://choishingwan.github.io/PRS-Tutorial/base/)

gunzip -c ADrisk.txt.gz |\
awk 'NR==1 || ($8 > 0.9) {print}' |\
gzip  > fAnyPE.txt.gz


# Rearrange GWAS summary statistics file to select the columns of the following variables: SNP, A1, A2, OR (or BETA), P

cat ADrisk.txt | awk '{print $6, $4, $5, $13, $8}' > pADrisk.txt

# Remove duplicate SNPs

# Generate a file with duplicate SNPs
gunzip -c pfADrisk.txt.gz |\
awk '{ print $1}' |\
sort |\
uniq -d > duplicated.snp

# Remove duplicate SNPs
gunzip -c pfADrisk.txt.gz  |\
grep -vf duplicated.snp |\
gzip - > pfADrisk_nodup.txt.gz
```

 https://doi.org/10.3390/genes16101128

```
# Generate training effect sizes using PRScs-AUTO method

python3 PRScs.py --ref_dir=/data/ldblk_1kg_eur --bim_prefix=/data/PRScs_master/WGS_Omni25_BIN_wo_ConsentsIssues.qc --sst_file=/data/PRScs_master/pADrisk_nodup.txt --n_gwas=127966 --n_iter=2000 --n_burnin=1000 --out_dir=/data/PRScs_master

# Concatenate output files

cat PRScs_master_pst_eff_a1_b0.5_phiauto_chr*.txt > ADrisk_pst_eff_a1_b0.5_phiauto.txt

# Add the p value from the raw sumstats file to the concatenated output

cat SNPsubset_sorted.txt | awk '{print $2}' > P_values.txt
paste ADrisk_pst_eff_a1_b0.5_phiauto.txt P_values.txt > ADrisk_final.txt

# Generate PRS with PRSice using PRScs output

Rscript PRSice.R --out ADNI.AD.prscs \
--prsice PRSice_linux \
--base ADrisk_final.txt --beta \
--target WGS_Omni25_BIN_wo_ConsentsIssues.qc --no-clump \
--bar-levels 0.0001,0.001 --fastscore --score avg --no-regress
```
